# UltraSEQ, a Universal Bioinformatic Platform for Information-Based Clinical Metagenomics and Beyond

**DOI:** 10.1128/spectrum.04160-22

**Published:** 2023-04-11

**Authors:** Bryan T. Gemler, Chiranjit Mukherjee, Carrie Howland, Patrick A. Fullerton, Rachel R. Spurbeck, Lindsay A. Catlin, Anthony Smith, Angela T. Minard-Smith, Craig Bartling

**Affiliations:** a Battelle Memorial Institute, Columbus, Ohio, USA; American Type Culture Collection

**Keywords:** bioinformatics, clinical, diagnostics, metagenomics, next-generation sequencing

## Abstract

Applied metagenomics is a powerful emerging capability enabling the untargeted detection of pathogens, and its application in clinical diagnostics promises to alleviate the limitations of current targeted assays. While metagenomics offers a hypothesis-free approach to identify any pathogen, including unculturable and potentially novel pathogens, its application in clinical diagnostics has so far been limited by workflow-specific requirements, computational constraints, and lengthy expert review requirements. To address these challenges, we developed UltraSEQ, a first-of-its-kind accurate and scalable metagenomic bioinformatic tool for potential clinical diagnostics and biosurveillance utility. Here, we present the results of the evaluation of our novel UltraSEQ pipeline using an *in silico*-synthesized metagenome, mock microbial community data sets, and publicly available clinical data sets from samples of different infection types, including both short-read and long-read sequencing data. Our results show that UltraSEQ successfully detected all expected species across the tree of life in the *in silico* sample and detected all 10 bacterial and fungal species in the mock microbial community data set. For clinical data sets, even without requiring data set-specific configuration setting changes, background sample subtraction, or prior sample information, UltraSEQ achieved an overall accuracy of 91%. Furthermore, as an initial demonstration with a limited patient sample set, we show UltraSEQ’s ability to provide antibiotic resistance and virulence factor genotypes that are consistent with phenotypic results. Taken together, the above-described results demonstrate that the UltraSEQ platform offers a transformative approach for microbial and metagenomic sample characterization, employing a biologically informed detection logic, deep metadata, and a flexible system architecture for the classification and characterization of taxonomic origin, gene function, and user-defined functions, including disease-causing infections.

**IMPORTANCE** Traditional clinical microbiology-based diagnostic tests rely on targeted methods that can detect only one to a few preselected organisms or slow, culture-based methods. Although widely used today, these methods have several limitations, resulting in rates of cases of an unknown etiology of infection of >50% for several disease types. Massive developments in sequencing technologies have made it possible to apply metagenomic methods to clinical diagnostics, but current offerings are limited to a specific disease type or sequencer workflow and/or require laboratory-specific controls. The limitations associated with current clinical metagenomic offerings result from the fact that the backend bioinformatic pipelines are optimized for the specific parameters described above, resulting in an excess of unmaintained, redundant, and niche tools that lack standardization and explainable outputs. In this paper, we demonstrate that UltraSEQ uses a novel, information-based approach that enables accurate, evidence-based predictions for diagnosis as well as the functional characterization of a sample.

## INTRODUCTION

Traditional clinical microbiology-based diagnostic tests rely on targeted methods that can detect only one to a few preselected organisms by the use of molecular methods (e.g., quantitative PCR [qPCR] or antigen detection methods) or slow, culture-based methods. Although widely used today, these methods have several limitations, especially for infections caused by more than one etiological agent, novel pathogens, and unculturable organisms. Furthermore, these tests are often singleplex, requiring multiple tests to be run prior to diagnosis. Due to these limitations, the rate of unknown etiology of infection has been reported to be >50% for diseases such as pneumonia and encephalitis (1, 2). This rate of unknown etiology is corroborated by our initial analysis of commercial health claims of 2021 International Classification of Disease (ICD) codes, which suggests that despite more than $5 billion being charged in 2021, as many as 80% of pneumonia cases (average of inpatients and outpatients) were coded with an “unspecified organism” ICD code (https://www.definitivehc.com/) (C. Bartling, unpublished data).

Massive developments in sequencing technologies have made it possible to apply metagenomic methods to clinical diagnostics, which may alleviate the issues described above. Metagenomics offers a hypothesis-free approach to identify any pathogen, including unculturable and potentially novel pathogens, in a massively multiplexed assay that requires little to no wet-laboratory assay development for new etiological agents. The approach is largely unbiased, can work well with very-low-biomass samples, can often replace invasive sampling regimes, and, ultimately, can lead to better patient outcomes and reduce antibiotic misuse by the quick, accurate determination of the cause of the disease (3). Clinical metagenomics is rapidly moving from research to the clinic, with commercial offerings for diseases such as sepsis, respiratory disease, and meningitis/encephalitis (3). However, current offerings are limited to a specific disease type or sequencer workflow and/or require laboratory-specific controls, thus limiting their widespread adoption.

The limitations associated with current clinical metagenomic offerings result from the fact that the backend bioinformatic pipelines are optimized for the specific parameters described above, resulting in an excess of unmaintained, redundant, and niche tools that lack standardization and explainable outputs. For pathogen identification, several different classifiers exist, including those that leverage alignment-based (both nucleic acid and protein) and k-mer-based approaches (4, 5). Classification can be provided on the individual-sequence level (binning) as well as the whole-data-set level (profiling). While k-mer-based methods are fast, efficiency must be weighed against the optimization of k-mer sizes with each database update as well as the lack of granularity in predictions due to exact matching (i.e., important variable sequence information relevant to clinical settings may be lost [6]). Thus, k-mer-based approaches can be highly specific but suffer from low sensitivity, particularly for the identification of novel pathogens such as viruses with high mutation rates (7). Most clinical metagenomic tools have adopted rapid alignment routines to achieve high sensitivity. However, such routines often result in the overidentification of organisms in samples, many of which may be false-positives (FPs). Tools may overcome these high false-positivity rates by using background subtraction methods and thresholding on various settings that are optimized for each specific workflow. Often, conservative predictions are made at higher taxonomic levels (e.g., genus level) that are less informative for clinical applications.

To address all of these challenges, we developed UltraSEQ, a first-of-its-kind metagenomics-based tool with diagnostic potential that is accurate and scalable and provides an information-based approach for clinical research applications. As described above, many bioinformatic routines for pathogen identification suffer from high false-positivity rates, the inability to identify organisms not included in their reference database, and either complexity associated with the interpretation of results or nonexplainable “black box” answers. In contrast, UltraSEQ uses a novel, information-based approach that leverages a fast aligner that can handle both DNA and protein databases to make sample-level predictions (including taxonomic profiling) at the most specific taxonomic levels possible given the information for the sample and the database(s) used. UltraSEQ was built from the ground up to make predictions for regions of sequences (including taxonomic binning), full sequences, and collections of sequences (i.e., a sample) without complicated user settings and the necessity for background data set subtraction. This novel approach enables accurate, evidence-based predictions for diagnosis as well as the functional characterization of a sample, including virulence factor and antibiotic resistance (AbR) profiles. Predictions are backed by our curated database that provides end users with additional contextual metadata of predictions, such as whether the identified pathogen typically or rarely causes disease or is a potential normal flora contaminant as well as other user-defined characteristics. In our previous work, we described the development of this database (8), which uses a function-centric approach to distinguish between pathogenic and nonpathogenic organisms with increased confidence over the state of the art. Here, we expand on this approach to leverage this database by building the flexible UltraSEQ bioinformatic platform and demonstrate its potential utility for clinical metagenomic applications that go beyond simple taxonomic predictions. We demonstrate UltraSEQ’s ability to handle a variety of different sample types, sequencing platforms, and laboratory workflows and UltraSEQ’s superior performance compared to several other bioinformatic platforms.

## RESULTS

**FIG 1:**
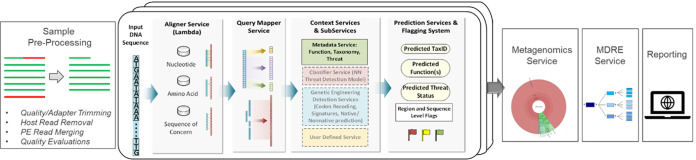
Graphical representation of the UltraSEQ pipeline. Reads are optionally preprocessed and aligned to reference databases, and distinct regions within each read are identified. Taxonomy and functional annotations are assigned at the read level and are subsequently combined into sample-level taxonomy profiles by the metagenomics services. The metagenomics diagnostics rules engine (MDRE) service is specific for clinical diagnostics and uses decision trees to identify species and their virulence factors in the sample that cause disease before the reporting of all results. PE, paired end; NN, neural net.

The overall schema of the UltraSEQ pipeline is presented in Fig. 1. Reads are first optionally preprocessed to ensure high-quality data. Each read is then aligned to multiple reference databases, and the query mapper server identifies regions in each read. Context services assign taxonomy and functional annotations to each region of each read. The metagenomics module combines information from all reads into a sample-level taxonomy profile. The metagenomics diagnostics rules engine (MDRE) service analyzes and prioritizes results for clinical diagnostics. Results are subsequently reported using UltraSEQ’s cloud-deployed Web application.

To comprehensively evaluate UltraSEQ’s ability to accurately taxonomically profile metagenomic samples with the goal of data-set-agnostic diagnostic application, we applied a 3-step evaluation approach. In step 1, we evaluated the specificity and sensitivity of UltraSEQ using an *in silico*-synthesized metagenome consisting of genomic DNAs from 22 different microbial species, including bacteria, fungi, viruses, and humans. In step 2, we evaluated UltraSEQ against a mixed microbial community data set to measure real-world performance. Finally, in step 3, we evaluated UltraSEQ using publicly available clinical metagenomic data sets to compare our results with clinical microbiology test results as well as those from other metagenomic pipelines. The results for each of these comparisons are presented below.

### *In silico* data sets.

The information-theory-based approach combined with machine-learning algorithms in UltraSEQ allows a highly tunable final report, ranging from very conservative reporting, where false positives are minimized, to more permissive settings, which allow more false positives while ensuring minimal false negatives (FNs). Using our *in silico*-synthesized metagenomic sample, we extensively tested the different parameters and found that the tuning of UltraSEQ’s metagenomics service module (metagenomic module) had the most consequential effect on taxonomic assignment reporting. Within the metagenomic module, this tuning is achieved by altering the user-settable k-means clustering centroid distance tolerance threshold, referred to here as the metagenomic clustering threshold (MCT) (see File S1A in the supplemental material). Our results show that a minimum MCT of 0.5 results in the lowest FN counts for the data set tested, with a very marginal increase in the FP count (Table 1). Using an MCT of 0.5, the 4 “false-positive” species detected, all at very low relative abundances, were Aspergillus fumigatus, Lacticaseibacillus paracasei, Prevotella intermedia, and Streptococcus equinus, which indicates reads being assigned from the true-positive (TP) species Aspergillus niger, Lactobacillus casei, Prevotella denticola, and Streptococcus mutans, respectively.

**TABLE 1:** Effect of the metagenomic clustering threshold parameter on the specificity and sensitivity of UltraSEQ’s taxonomic assignment reporting for our *in silico*-synthesized sample

MCT	No. of samples		False-negative result(s)
	True positive	False positive	
0.1	18	0	Human herpesvirus 1, Listeria monocytogenes, Zika virus
0.2	19	2	Human herpesvirus 1, Zika virus
0.3	19	2	Human herpesvirus 1, Zika virus
0.4	19	2	Human herpesvirus 1, Zika virus
0.5	21	4	None
0.75	21	4	None
0.8	21	4	None
0.9	21	4	None
1.0	21	4	None

**FIG 2:**
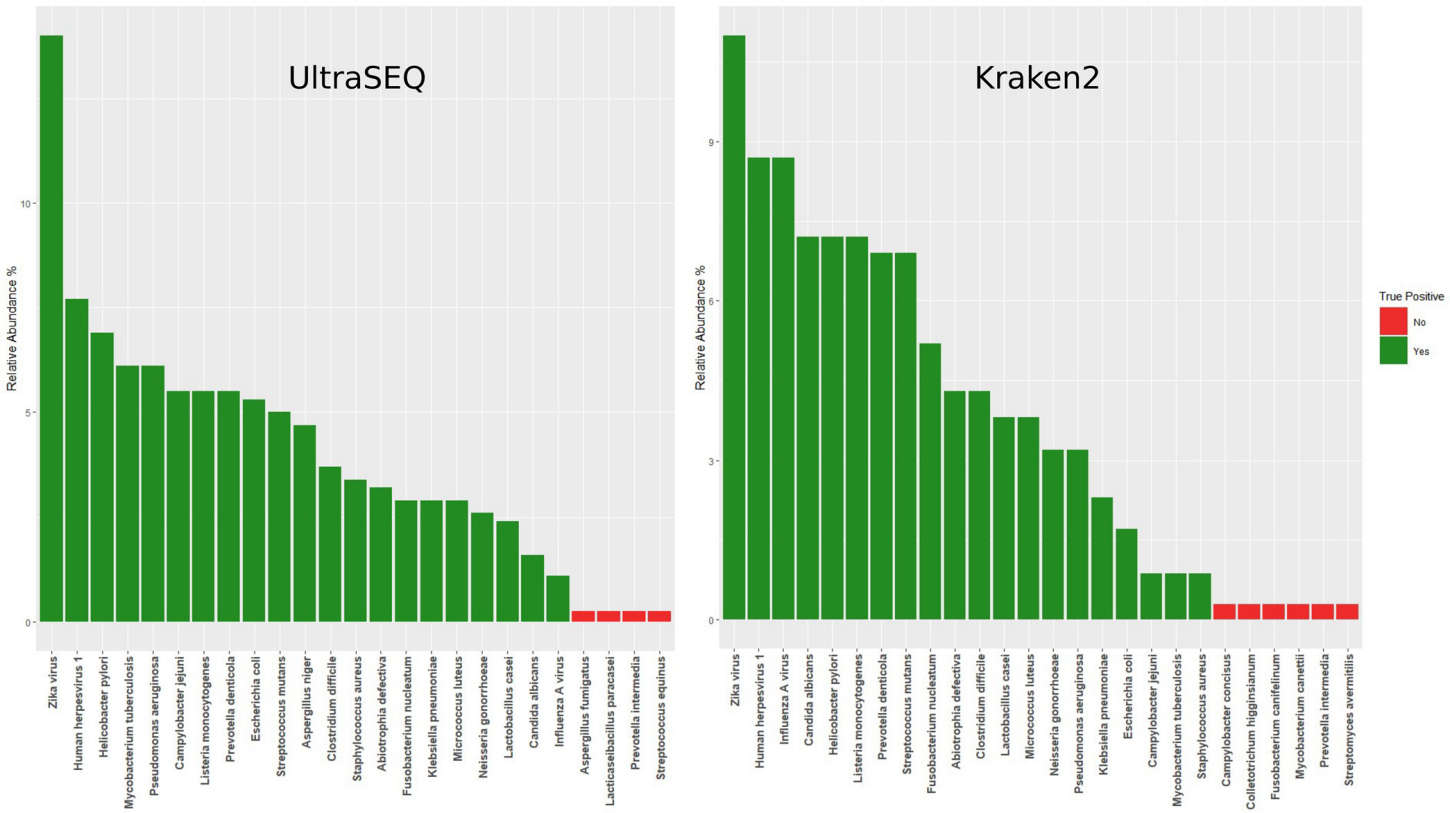
Bar plots showing the relative abundance of each species detected from the synthetic metagenome sample using UltraSEQ (left) and Kraken2 (right).

To compare the taxonomic classification results obtained using UltraSEQ’s metagenomic module with the those of current gold standards in k-mer mapping-based taxonomy classification tools, we processed the synthetic metagenome sample with Kraken2 using default settings against the PlusPf database provided by the Langmead Lab (https://benlangmead.github.io/aws-indexes/k2) (9). Bar plots comparing the taxonomy results for UltraSEQ (MCT of 0.5) and Kraken2 are presented in Fig. 2. While the relative abundances for the false-positive samples were low (<1%) with both methods, Kraken2 had a higher number of false positives (6) and also failed to detect A. niger in the sample.

### Mixed microbial community data sets.

To further test the metagenomics service with real biological sequences, a mixed microbial community sample, sequenced using both the Nanopore and Illumina platforms as described in Materials and Methods, was processed using UltraSEQ. Based on our findings with the *in silico* data set, these samples were also processed with an MCT of 0.5. UltraSEQ correctly predicted the presence of all 10 species for both the long- and short-read data sets with relative abundance values close to the expected values (Table 2). The highest false-positivity rate detected in both data sets was for Bacillus spizizenii, which is very closely related to the expected species, Bacillus subtilis. For both data sets, any false-positive species detected were present at a <0.4% relative abundance compared to the expected species. These results, and those observed for the synthetic metagenome, led us to apply a domain-specific abundance filter to UltraSEQ’s results for subsequent application in clinical metagenomics.

**TABLE 2:** UltraSEQ correctly identifies all organisms in a mock microbial community data set

Species	Expected relative abundance (%)	Observed relative abundance (%)	
		Illumina data set	Nanopore data set
Bacillus subtilis	12	11.9	8.9
Enterococcus faecalis	12	12.0	9.6
Escherichia coli	12	12.4	10.5
Lactobacillus fermentum	12	9.0	8.9
Listeria monocytogenes	12	12.9	14.6
Pseudomonas aeruginosa	12	14.7	8.5
Salmonella enterica	12	12.8	11.6
Staphylococcus aureus	12	10.4	18.8
Cryptococcus neoformans	2	0.5	1.5
Saccharomyces cerevisiae	2	1.8	2.1

### Clinical data sets.

In total, 10 different sets of metagenomic data sets, encompassing 407 clinical comparisons from 216 samples, were analyzed. These data sets spanned a range of clinical sample types (cerebrospinal fluid [CSF], nasal, and oral, etc.), disease types (respiratory disease and encephalitis/meningitis), sequence types (RNA and DNA), and sequence generators (Illumina, IonTorrent, and Nanopore). For each data set, we evaluated UltraSEQ’s diagnostic capability by comparing the results from metagenomic data sets of clinical samples to microbiological results for the same samples. For this analysis, we calculated the positive percent agreement (PPA), negative percent agreement (NPA), and accuracy (ACC) of UltraSEQ with the publicly available sequence data sets or in-house data sets. Overall, UltraSEQ demonstrated 86% PPA, 97% NPA, and 91% accuracy across all 10 sets of data, demonstrating UltraSEQ’s utility across the wide range of data sets tested. For each data set, we further compared the performance of UltraSEQ to those of other informatic tools with the above-mentioned metrics as well as additional metrics (antibiotic resistance profiles and usability, etc.), as detailed below. In contrast to other pipelines, no background data sets were used to generate UltraSEQ results, which is the most direct comparison to clinical microbiological results. All results are shown in File S3, with each study in a separate tab.

**FIG 3:**
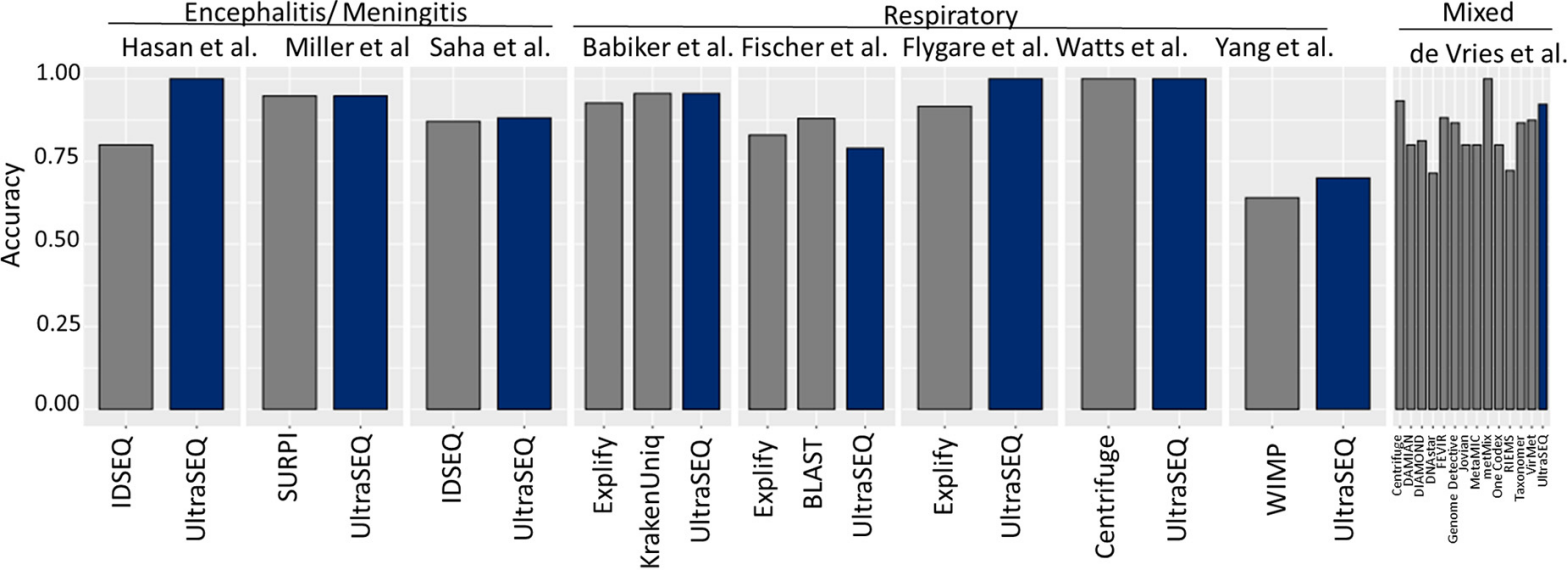
Comparison of UltraSEQ clinical diagnostic performances.

Figure 3 shows a summary of UltraSEQ’s clinical diagnostics accuracy against other bioinformatic tools noted for each case study comparison.

### Case study 1 (Miller et al.).

A study by Miller et al. (10) (BioProject accession number PRJNA516289) investigated the use of both RNA sequencing (RNAseq)-based and DNA sequencing (DNAseq)-based metagenomics to diagnose encephalitis/meningitis, and the SURPI pipeline was used to make organism calls. For this study, the 90 data sets derived from 76 samples without a high host background as reported by those authors were run for comparison. The results for these 90 data sets were compared to the results of 173 clinical and/or confirmatory tests (positive or negative). Overall, UltraSEQ performed the same in terms of accuracy (95%) compared to Miller et al.’s pipeline across all 173 clinical tests (Table SF1). UltraSEQ performed better than Miller et al.’s pipeline in terms of NPA (100% for UltraSEQ) but not PPA (85% for UltraSEQ compared to 86% for Miller et al.’s pipeline). Importantly, UltraSEQ reported zero false positives even without the use of a background data set subtraction method. Across the various categories tested, UltraSEQ performed better for fungi and bacteria, worse for DNA viruses and RNA viruses, and the same for parasites (Table SF1). For fungi, Miller et al.’s one false negative was due to the lack of identification of Sporothrix schenckii, which UltraSEQ identified as a pathogen that can cause encephalitis/meningitis (11). For bacteria, Miller et al. identified *Bacillus* species as a false positive in one sample, whereas UltraSEQ did not identify any *Bacillus* species. For DNA viruses, two false negatives were due to reported coinfections by two different types of herpesviruses. For RNA viruses, the two false negatives reported by UltraSEQ that were not reported by Miller et al. were due to only 2 reads or fewer being identified as the true virus, which did not enable high-enough confidence in UltraSEQ reporting a positive result.

### Case study 2 (Saha et al.).

A study by Saha et al. (12) (BioProject accession number PRJNA516582) also investigated the use of metagenomic RNA sequencing for the diagnosis of encephalitis/meningitis. Overall, UltraSEQ performed slightly better than those authors’ pipeline for the PPA and ACC metrics, with one fewer false-negative result (Table FS2). Not surprisingly, a general correlation between qPCR cycle threshold (*C_T_*) values and positive results was observed. For the seven cases where qPCR values were relatively high and both UltraSEQ and Saha et al. reported FNs, UltraSEQ detected Streptococcus pneumoniae in 6/7 (86% of the cases). However, all were below the 1% threshold used to make a positive call. If the criteria for S. pneumoniae were loosened from these default criteria to require only a 0.3% relative abundance, 3 additional FNs would become TPs (88% PPA for all samples). UltraSEQ reported no false positives for all samples (100% NPA), but a direct comparison of NPA values to those of Saha et al. could not be performed since those authors did not report false positives.

### Case study 3: CSF_metagenomics from idseq.net (Hasan et al.).

The encephalitis/meningitis data set of Hasan et al. (13) was selected to compare UltraSEQ to CZ ID (formerly IDseq) (14). Since most of the 20 available samples were true negatives (TNs), 5 samples with confirmed truth tests (i.e., comparative molecular tests) were selected for a proof-of-concept comparison. For the 5 samples analyzed, UltraSEQ successfully called all 5 correctly (100% accuracy), whereas Hasan et al. called 4/5 correctly (80% accuracy). In the one case where Hasan et al. called a false positive, UltraSEQ did not identify the pathogen (Streptococcus parasanguinis). To further demonstrate the differences between UltraSEQ and CZ ID, results from the analysis of sample CW322 by UltraSEQ (Table SF3) and CZ ID (Fig. SF1 and SF2) were compared. UltraSEQ identified only two species and automatically filtered out other species with low confidence, without requiring user input. In contrast, CZ ID identified many bacterial and viral genera (Fig. SF1), and within each genus, several species were identified. For example, Fig. SF2 shows partial results for *Neisseria* in which 22 species were identified.

### Case study 4 (Fischer et al.).

In a previous study by Fischer et al. (15) (BioProject accession number PRJEB7888), Illumina RNAseq reads from 24 different respiratory disease patients were evaluated in comparison to qPCR influenza clinical data. The UltraSEQ results were compared to the results of Fischer et al.’s in-house BLAST pipeline and Explify. For this comparison, positive detection was defined as the identification of influenza virus, and negative detection was considered a lack of influenza virus detection. All pipelines demonstrated 100% NPA but varied in their PPAs, with UltraSEQ failing to identify influenza virus in one more and two more cases than with Fischer et al.’s and Explify’s pipelines, respectively (Table SF4 and File S3).

### Case study 5 (Watts et al.).

A study by Watts et al. (16) (BioProject accession number PRJNA554856) included samples from two patients with ventilator-associated pneumonia (VAP), collected on day 1 and day 3, for a total of 4 samples sequenced using the Thermo Fisher Scientific IonTorrent platform. For patient 1, UltraSEQ identified Staphylococcus aureus only on day 3, which is in good agreement with the culture results and Watts et al.’s results (File S3). For patient 2, UltraSEQ identified S. aureus and Klebsiella aerogenes on day 1, which is in good agreement with the culture results and those authors’ results. Other positive results were identified as well, including Prevotella melaninogenica, Pseudomonas aeruginosa, and human alphaherpesvirus for patient 1.

### UltraSEQ for antibiotic resistance profiling.

In addition to pathogen identification, UltraSEQ provides antibiotic resistance (AbR) profiles based on the presence of genes that are known to cause resistance to various antibiotics, as described in Materials and Methods. The results of AbR profiling for S. aureus from the sample under SRA accession number SRR9693434 are shown in Table SF5, which are consistent with the microbial culture results and those authors’ results using ResFinder (17). UltraSEQ identified a profile consistent with resistance of S. aureus to methicillin through the detection of the *mecA* gene. The *mecA* gene was not detected in any other samples, consistent with the authors’ report. Other potential resistance genes were identified as well, but no other phenotype testing was performed, and thus, no comparative conclusions can be drawn.

### Case study 6 (Yang et al.).

A previous study by Yang et al. (18) (BioProject accession number PRJNA554461) investigated the use of clinical metagenomics using an Oxford Nanopore MinION sequencer for patients with VAP. Here, the UltraSEQ results were compared to the results of Oxford Nanopore’s What’s in My Pot (WIMP) workflow, as reported by Yang et al. As shown in Table SF6, UltraSEQ’s results demonstrated a much higher NPA due to 4 fewer false positives but a slightly lower PPA due to one more false negative. For the 4 culture-negative pneumonia cases (cases 10 and 12 to 14) and the 8 control cases (cases 15 to 22), the UltraSEQ results were consistent with those authors’ (WIMP) results, with some exceptions, as detailed in the scores tab of File S3. For the 10 culture-positive pneumonia cases (cases 1 to 9 and 11), UltraSEQ identified at least one concordant pathogen in 8/10 cases (discordant for cases 7 and 9); in comparison, those authors identified the concordant pathogen in 9/10 cases. However, those authors identified 2 false positives for both case 1 and case 4, which UltraSEQ correctly did not report. For case 1, UltraSEQ identified the FP organisms, but both fell below the abundance threshold described in Materials and Methods. For case 4, UltraSEQ did not identify the 2 FPs at any abundance.

### Antibiotic resistance profiling.

To further test UltraSEQ’s ability to identify AbR profiles based on genotypes, the UltraSEQ results were compared to the phenotype results reported by Yang et al., who used ResFinder (17) (Table SF7). In general, the UltraSEQ results showed excellent agreement with the phenotypic results, and the results provide a direct interpretation of genotypes (i.e., UltraSEQ automatically interprets antibiotic resistance based on CARD hits, whereas Yang et al. required manual interpretation based on hits for various genes). Specifically, for 4 cases, the phenotypes of antibiotic resistance to 11 antibiotics were identified using culture profiling; of these 11 antibiotics, UltraSEQ identified pathogen-specific evidence (i.e., the resistance genes were likely derived from the identified pathogen) for resistance to 7 of those antibiotics or classes of antibiotics. For 3 of the remaining 4 antibiotics (all fluoroquinolone antibiotics), UltraSEQ identified pathogen-agnostic evidence (i.e., the gene was not identified to be associated with the identified pathogen); for the remaining 1 of 4, UltraSEQ identified pathogen-specific evidence of resistance to a closely related antibiotic (different type of β-lactam antibiotic).

### Case study 7 (Flygare et al. and Graf et al.).

Previous studies by Flygare et al. and Graf et al. (19, 20) (BioProject accession number PRJEB13360) investigated the use of Illumina RNA sequencing of 24 upper respiratory tract samples for the diagnosis of diseases caused by respiratory viruses. No negative controls were included in these studies, and all results were compared to the results of PCR tests. For these 24 samples, Flygare et al. reported an average of 95% sensitivity (positive predictive agreement) for their Protonomer module of Taxonomer software (average as reported in Fig. 3 in reference 19). In contrast, UltraSEQ identified the correct virus in all 24 samples (100% PPA). Since the individual viruses that UltraSEQ identified in each of the 24 samples were not reported by Flygare et al., we performed a head-to-head comparison of the results of UltraSEQ for a subset of the samples to the results of Explify, the software developed from Taxonomer software (21). For the 12 samples analyzed, Explify correctly identified 11/12 (92% PPA), whereas UltraSEQ identified 12/12 (100% PPA) (File S3). For SRA accession number ERR1360082, Explify failed to identify enterovirus B. The results for all other samples were very similar.

### Case study 8 (Babiker et al.).

A study by Babiker et al. (22) (BioProject accession number PRJNA634356) investigated Illumina RNA sequencing data sets from nasopharyngeal (NP) swabs for the diagnosis of severe acute respiratory syndrome coronavirus 2 (SARS-CoV-2) infection. Pathogen detections were compared to the results for SARS-CoV-2 reverse transcription-PCR (RT-PCR)-positive patients (*n* = 45) and RT-PCR-negative subjects (*n* = 30), including one viral coinfection confirmed by RT-PCR. We compared UltraSEQ’s results to those of Babiker et al., who used KrakenUniq (23), and a subset was also run using Explify. Molecular and confirmation testing used by those authors detected viruses such as influenza virus, respiratory syncytial virus (RSV), parainfluenza virus, human metapneumovirus (hMPV), rhinovirus, and/or human coronaviruses (HCoVs). Thus, a detection by any pipeline was scored positive only if it detected one of the above-mentioned viruses and the specific test was run for that sample. As compiled in Table SF8 and File S3, UltraSEQ’s results demonstrated a higher PPA than both pipelines due to no false negatives. In contrast, Babiker et al. reported 1 FN (GA-EHC-084F), and the use of Explify software resulted in 3 FNs. For FPs, UltraSEQ identified two, in contrast to one FP reported by Babiker et al. and zero by Explify. Taken together, these results demonstrate that UltraSEQ has an overall accuracy of 96% for this data set, compared to 96% and 93% for Babiker et al.’s pipeline and Explify, respectively.

### Case study 9 (de Vries et al.).

A previous study by de Vries et al. (24) compared 13 different bioinformatic pipelines for the diagnosis of respiratory disease and encephalitis from metagenomic data sets. For this data set, UltraSEQ showed a 100% positive predictive value (PPV) (no false positives) and a 92% PPA (sensitivity), as shown in Table SF9. This PPA value was higher than those for 10 of the other 13 pipelines, but only 1 (metaMix) of the 3 pipelines that had higher PPA values had an equivalent PPV. The negative predictive value (NPV) or accuracy was not calculated here since no true-negative samples were included in the data set.

### Case study 10: in-house COVID-19 data set.

To further test UltraSEQ’s ability to diagnose respiratory disease with a different sample type (saliva), we generated and analyzed 8 RNAseq metagenomic data sets from coronavirus disease 2019 (COVID)-19-positive patients (BioProject accession number PRJNA856680). These results were compared to gold-standard PCR tests (File S3). For 6 of the 8 samples, we correctly identified SARS-CoV-2 (75% true-positivity rate). No other pathogens (except for oral/throat bacterial contaminants) were identified. For the two false negatives, no reads were identified that aligned to SARS-CoV-2, and the lack of detection did not correlate with the qPCR results, suggesting that the lack of detection was due to a lack of a signal in the data set and not due to UltraSEQ.

## DISCUSSION

Applied metagenomics is a powerful emerging capability enabling the untargeted detection and characterization of pathogens, reducing the time window for and enhancing the identification of emerging and traditional threats for both clinical and biosurveillance applications. Realizing the potential for metagenomics requires bioinformatic solutions that not only can keep pace with scientific discovery but also are scalable, accurate, and science backed. While clinical metagenomic tools have been developed and validated for specific applications, their utility is limited by workflow-specific requirements, computational constraints, lengthy expert review, and stagnant databases. For example, SURPI has been validated for CSF samples/encephalitis using Illumina reads (10), Explify has been validated for respiratory samples/disease using Illumina reads (19, 20), and Karius has been validated for cell-free DNA/sepsis from Illumina reads (25), but each of these requires specific wet-laboratory workflows, instrumentation, and background controls. In parallel, numerous metagenomic tools have been developed for taxonomic identification with potential utility for surveillance, such as CZ ID (14) and What’s in My Pot (WIMP) (26), but such tools will likely not have widespread clinical application until they are validated across various use cases.

Here, we present results for the evaluation of our novel UltraSEQ pipeline using *in silico* data sets, mock microbial community data sets, and publicly available clinical data sets across a wide range of applications. For the *in silico* data set, UltraSEQ successfully detected all 21 species across the tree of life, with fewer false positives than with Kraken2 (Fig. 2). UltraSEQ also detected all 10 bacterial and fungal species in the mock microbial community data set sequenced by both the Illumina and Nanopore platforms (Table 2). Finally, the clinical data sets contained samples from different infection types (encephalitis, meningitis, and other respiratory diseases), comprised of both short-read (Illumina and IonTorrent) and long-read (Nanopore) sequencing data in both RNAseq and DNAseq formats, and represented both sterile (e.g., spinal fluid and blood) and “dirty” (e.g., saliva and nasal) sample types. Here, UltraSEQ’s pathogen detection accuracy was the same as or better than those of the comparable bioinformatic tools used in the studies for seven of the nine clinical data sets (Fig. 3). UltraSEQ analyzed the set of diverse samples, identified pathogens, and achieved an overall accuracy of 91%, even without requiring data-set-specific configuration setting changes, background data set subtraction, or prior sample information. We also demonstrated UltraSEQ’s ability to provide accurate antibiotic resistance and virulence factor genotypes that are consistent with phenotypic results with a limited data set. Future work will focus on a more extensive validation of this feature.

**FIG 4:**
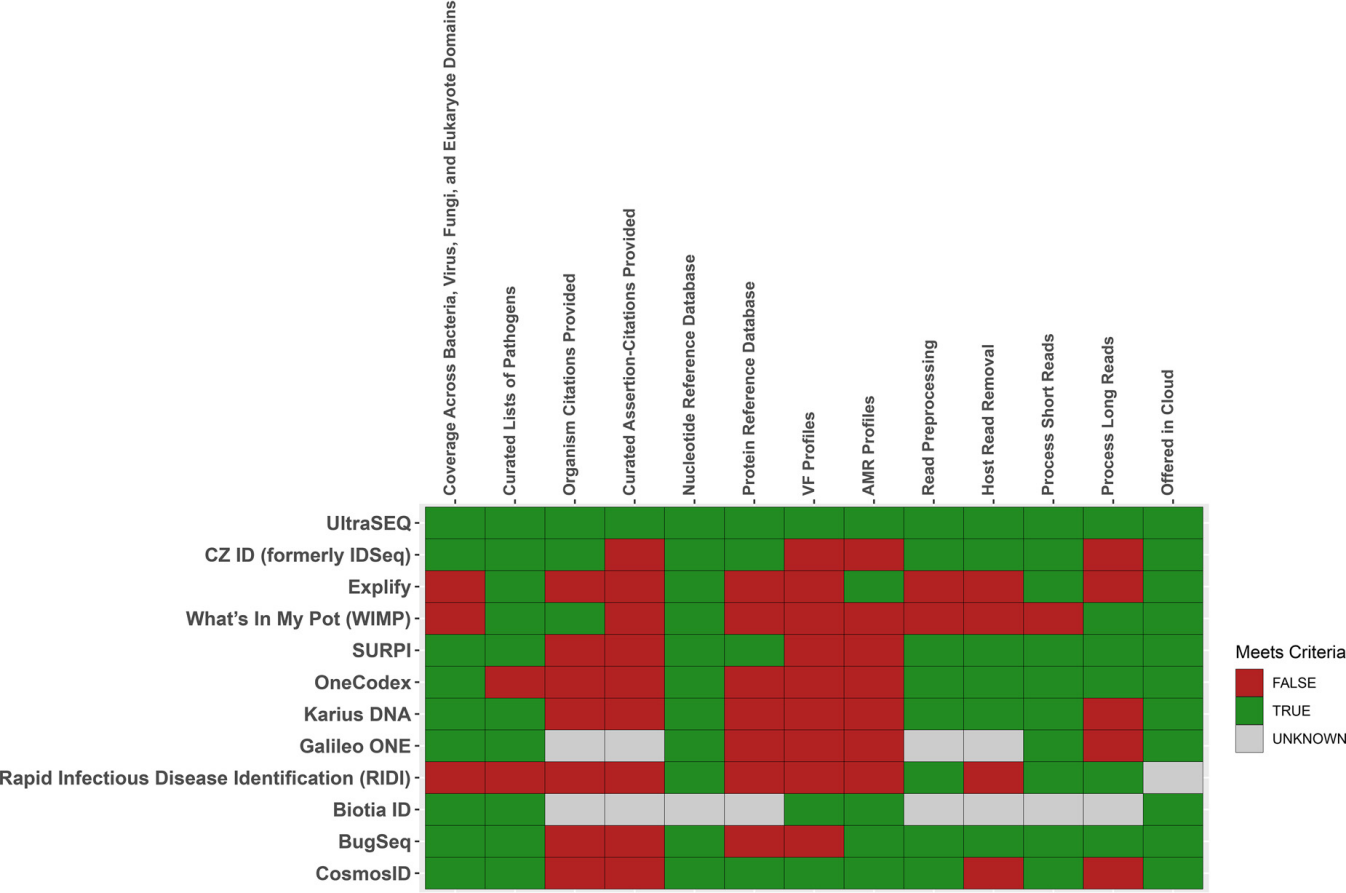
Comparison of UltraSEQ to commonly used bioinformatic tools for clinical diagnostics or surveillance (https://www.cosmosid.com) (10, 14, 21, 26, 27, 38–48). VF, virulence factor.

Taken together, the above-described results demonstrate that Battelle’s UltraSEQ platform offers a transformative approach for microbial and metagenomic sample characterization, employing biologically informed detection logic, deep metadata, and a flexible system architecture for the classification and characterization of taxonomic origin, gene function, and user-defined functions, including disease-causing infections. A highly curated pathogen and virulence factor database underpins the UltraSEQ analytics engine and enables the rapid, accurate, and explainable detection and characterization of pathogens. In addition to the results shown here, we further discuss the important features of UltraSEQ software compared to both the benchmarked software described in Results and additional well-known software used for clinical diagnostics and/or surveillance research (Fig. 4). While each of the 12 pipelines provides advantages and disadvantages, UltraSEQ’s curated databases, logic-based approach, and modularity provide accurate predictions across a wide range of data set types with best-in-class comprehensiveness of reference database coverage, science-backed annotations, and flexibility.

### Comprehensiveness.

Metagenomic-based identification of pathogens relies on matching query sequences to sequences in a database, followed by prediction. To ensure the highest accuracy, maximal coverage across the tree of life is required to ensure that query reads are not incorrectly assigned. For example, nontypical parasites that can cause disease, such as *Trichinella* spp. (identified in the data set of Miller et al. [10]), can be identified only if they are included in subject databases. When comparing the 12 tools, UltraSEQ is one of the 9 tools that have taxonomy coverage across all relevant biological domains (bacteria, eukaryotes, viruses, and fungi). Of the three tools that do not have complete taxonomy coverage, Explify and WIMP are missing the eukaryote domain, and Rapid Infectious Disease Identification (RIDI) is missing virus, fungus, and eukaryote domains. Furthermore, UltraSEQ is one of only four tools that allow both nucleotide and protein reference databases to be included in the analysis pipelines, expanding the quantity of reference sequences that can be used to classify samples. In addition to taxonomic coverage, UltraSEQ’s databases include coverage and provide both virulence factor and antibiotic resistance genes, like two other tools (Fig. 4).

### Science-backed annotations.

While database coverage is important for pipeline performance, curation of the data contained in the database is perhaps even more important. All but two tools leverage curated lists of pathogenic organisms to enable the specific reporting of pathogens identified in a sample. For example, SURPI is backed by annotated lists of pathogens that cause encephalitis/meningitis, Karius is backed by lists of sepsis-causing pathogens, and Explify is backed by lists of respiratory-disease-causing pathogens, including granularity on phenotypic groups (normal flora and colonizers, etc.). UltraSEQ provides these curated lists as well but provides additional granularity, including the likelihood of the pathogen causing disease (e.g., immunocompromised), contaminant type (e.g., from laboratory reagents versus from native flora), and specific disease subtypes (e.g., ventilator-associated pneumonia), etc.

In addition, providing end users with human-readable text and primary references related to organisms, gene functions, and other information can minimize the burdens (such as review time) associated with bioinformatic predictions. Based on our research, UltraSEQ and at least two additional tools provide such text and citations. For example, CZ ID provides background information on organisms and provides Wikipedia citations, and WIMP provides background information on organisms at the genus level (27). However, to our knowledge, UltraSEQ is the only tool that provides curated assertions-citations of pathogens. Specifically, we provide end users with not only a short text rationale (assertion) for why the data are curated in a particular way but also the primary reference (e.g., PubMed identifier) in order to provide highly explainable and accurate results. For example, in the data set of Yang et al. (18), WIMP identified Stenotrophomonas maltophilia, Staphylococcus epidermidis, Pseudomonas aeruginosa, and Klebsiella pneumoniae for case 1, but only S. maltophilia was the likely disease-causing organism based on culture results. UltraSEQ identified S. maltophilia and S. epidermidis, but S. epidermidis could be eliminated because of its metadata (lack of association with VAP and known skin contaminant), and the reference for this annotation is provided to the end user.

### Flexibility and accuracy across data sets.

Like some other tools, UltraSEQ provides the functionality to both preprocess and remove host-derived reads. While most other tools perform read preprocessing, at least five do not include a host read removal step, which can increase the computation time. Furthermore, UltraSEQ and four other tools can process both short-read (e.g., Illumina and IonTorrent) and long-read (e.g., Nanopore and PacBio) sequencing data. Furthermore, UltraSEQ provides the additional flexibility of being deployed in different formats, including cloud deployment.

Most importantly, unlike the implementation of other tools described in this paper across the clinical case studies discussed, we have demonstrated that the potential implementation of UltraSEQ for clinical metagenomics for different sample types does not rely on parameters that are specific to read depth and background data set subtraction. For example, in their implementation of CZ ID, Saha et al. used specific filters that were dependent on read depth (e.g., NCBI NT reads of ≥10 and NCBI NR reads of ≥2, etc.) (12). For background subtraction, Miller et al. used a 10× threshold above the background sample (10) with their implementation of SURPI. Similarly, with their implementation of CZ ID, Hasan et al. and Saha et al. used (among other filters) an empirically derived Z score based on test sets of samples (12, 13). In contrast, UltraSEQ uses an information theory approach to calculate positive detections by utilizing the metagenomic module results and its own rules engine. Thus, the predictions are not dependent on read depth, and no background data set subtraction is required to achieve a high degree of specificity, allowing UltraSEQ to produce excellent results for any type of data set without *a priori* knowledge.

To further illustrate UltraSEQ’s lack of reliance on arbitrary depth thresholds and a background sample, we ran several data sets at various read depths and saw no degradation (or improvement) of UltraSEQ’s performance (data not shown). Furthermore, we noticed that Escherichia coli was a pervasive contaminant in many of these data sets, including the data sets of Hasan et al. and Miller et al. For these data sets, UltraSEQ also identified E. coli in most samples. Unlike the authors who used several custom user-defined filters and background samples to remove this contaminant, UltraSEQ correctly identifies E. coli as being present in the sample but provides the metadata label of biological contaminant. This context enables the end user, with minimal manual interpretation, to identify E. coli as a false positive and therefore eliminate it from the result without requiring background data. Overall, UltraSEQ made all of its predictions without a background sample, maintaining an accuracy comparable to or better than those in the studies that utilized a background sample subtraction analysis step. As for qPCR and other molecular assays, we suggest that background samples should be included as separate controls (and not used for background subtraction). Therefore, we believe that this feature of UltraSEQ will be a major benefit in its clinical metagenomics application since UltraSEQ does not require specific empirical testing to remove background signals and apply specific thresholds, thus expanding its utility across any sample type.

The future development of UltraSEQ will focus on improving its performance in classifying samples coinfected with multiple pathogens in the same domain (e.g., two viruses). For example, in the data set of Fischer et al. (15), UltraSEQ identified a false negative (SRA accession number ERR690513). For this sample, human alphaherpesvirus dominated the relative viral abundance maps (see Materials and Methods); thus, UltraSEQ likely did not report influenza virus in this case due to a low number of influenza virus reads relative to the number of human alphaherpesvirus reads. Another area of future development would be toward expanding and optimizing UltraSEQ’s ability to detect and classify emerging novel pathogens. Recent work in our laboratory analyzing novel SARS-CoV-2 demonstrated that UltraSEQ could identify SARS-CoV-2 as a SARS-CoV-1-related virus (data not shown). Furthermore, analytical models built from hazardous function signals (using the methodology reported in our recent publication [8]) demonstrated that SARS-CoV-2 clusters with other SARS-CoVs with similar host ranges and human receptor types. Future work will expand on these advances and improve UltraSEQ’s ability to characterize features of emerging pathogens of clinical and research significance.

## MATERIALS AND METHODS

### Overall UltraSEQ architecture and information flow.

The overall UltraSEQ pipeline is illustrated in Fig. 1. UltraSEQ handles various input file types and relies on high-quality sequence regions for downstream predictions. Each sequence has the option to go through all or some of the services described below, including a quality assurance preprocessing service, an aligner service, a query mapper service, context services, and prediction services. Collections of sequences can then undergo additional predictions, including taxonomic composition prediction (metagenomics module), a rules engine(s), and reporting services. A high-level summary of the services used in this study is included below; details of the services used in this study are provided in File S1A in the supplemental material, and other services not used during this study are described in File S1B (for the sake of completeness). UltraSEQ has a modular architecture with nonrestrictive deployment, including both local, high-performance computing cluster and cloud-based deployment. UltraSEQ is currently deployed on Amazon Web Services (AWS) in a secure, scalable computing environment complete with a graphical user interface to submit samples and download result reports and is available for use by contacting the corresponding author.

**(i) Preprocessing service.** UltraSEQ’s preprocessing routine includes steps to trim low-quality sequence regions, remove adapter sequences, remove duplicate sequences, (optionally) merge paired-end reads, and (optionally) remove host sequences (28–34). Different routines were applied for short-read (Illumina and IonTorrent) and long-read (Nanopore) data sets, as detailed in File S1A. Furthermore, to reduce cloud computing costs, enhance run times, and provide better comparisons across data sets, subsampling was optionally performed prior to the alignment service, as described in File S1A. This strategy enabled all samples to be run in minutes to hours (typically <2 h) using the computational resources described in File S1A.

**(ii) Aligner service.** Each sequence is rapidly aligned using LAMBDA2 version 1.9.5 (35) against selected databases, although other aligners can be used if desired. LAMBDA2 enables alignment against both protein and nucleotide databases, but the results for this study leveraged only protein databases since the inclusion of nucleotide databases did not improve the results based on an analysis of a subset of the data sets (data not shown); the inclusion of only protein databases also improved the run times and thus reduced the cloud computing costs. Specifically, for the analyses reported here, we used the publicly available UniRef100 protein database (built in April 2021 [https://www.uniprot.org/help/downloads]). UniRef100 provides coverage across the tree of life (pathogens and nonpathogens) and provides the foundation for taxonomic calls in UltraSEQ. In addition, we used Battelle’s Sequence of Concern (SoC) protein database, described previously (8). This database contains ~12,300 manually selected publicly available UniProt protein sequences that have been curated based on functions such as enabling antibiotic resistance, immune evasion, toxicity, and/or other threatening functions. The specific sequences are publicly available, but the exact compilation of the database is not published. Notably, the sequences in the SoC database are not required for taxonomic profiling as they are contained within UniRef100; they simply enable other downstream functionalities for UltraSEQ, including antibiotic resistance and virulence factor profiling. However, the SoC database further contains annotations of organisms (metadata) that are used in downstream UltraSEQ services (after taxonomic profiles are determined), as described below.

**(iii) Query mapper service.** The query mapper service maps regions within query sequences (i.e., portions of the query sequence) to identify high-quality-alignment regions as well as chimeric reads/out-of-context DNA sequences. For each query sequence, the positions of alignment starts and stops from high-quality alignments were compiled. A k-means clustering approach was used to identify positions in the query sequence with a high abundance of alignment starts/stops, which were subsequently used to identify regions of the query sequence.

**(iv) Context services and subservices.** Context services and subservices generate contextual information and pass information to downstream services. Information from the context services is passed to the prediction services and flagging system (rules engine), as described below.

**(v) Region-based prediction subservices.** For each region identified from the query mapper service, UltraSEQ predicts the taxonomy, function (gene ontology terms), and threat associated with the region. For this study, UltraSEQ’s sample-level taxonomy predictions (metagenomics module described below) were used for sample-level taxonomy calls, and UltraSEQ’s region-based function and threat prediction were not used. These calculations are provided in File S1B for the sake of completeness as they are useful for other use cases.

**(vi) Metagenomics service.** The metagenomics service provides the sample-level taxonomic composition based on the regions identified from reads processed by the query mapper service in 3 steps: (i) filtering out low-quality reads, (ii) scoring the remaining reads based on the information content of the reads, and (iii) predicting the taxonomic composition based on the scores. Sequence-region-level taxonomy predictions are associated with confidence scores that are based on the alignment quality. For each unique taxonomy identified in the sample, the confidence scores from the alignments from all query regions that are associated with it are assigned. A k-means clustering approach was used to identify taxonomies with high sample-level scores independently by taxonomy domain (*Bacteria*, *Archaea*, *Eukaryota*, and viruses). Detailed calculations are provided in File S1A.

**(vii) Rules engine service.** The rules engine service combines all of the above-described context and prediction services for regions, sequences, and samples using user-defined logic rules for rapid sequence triage. UltraSEQ currently has 4 default rules engines, but only the metagenomics diagnostics rules engine (MDRE) service was leveraged for this study (Fig. 1). The MDRE service enabled flexibility for diagnoses of different infectious disease types (e.g., respiratory disease and encephalitis/meningitis, etc.), as outlined in File S1E. In brief, the MDRE service is specific for clinical diagnostics and uses an array of decision trees to filter results based on curated data that are contained within the SoC database to down-select species and their virulence factors in the sample that cause disease.

**(viii) Reporting services.** UltraSEQ provides several reports, as described below.

*(a) Top-alignment report*. The top-alignment report is a simple report that provides the top alignments from each database (UniRef100 and SoC databases for this study) for each query sequence from the aligner and query mapper services. This report is used for downstream reports, as described below.

*(b) Taxonomy report*. The taxonomy report provides the alignments, annotated with the reference accession’s taxonomy identifier (TaxID), used by the metagenomics service and is used to determine the taxonomic composition of the sample. This report is used for downstream reports, as described below.

*(c) Default report*. The default report provides UltraSEQ’s region-level predictions for each sequence, including predictions for coarse threat functions (described above), taxonomy, gene ontology, and the sequence-level results of a rules engine(s) if applicable.

*(d) Sample report*. File S2 in the supplemental material provides a detailed description of the sample report. The “main-report” tab provides a list of all organisms identified from the above-described metagenomics service, the results associated with the identified organisms (relative abundance and number of SoCs identified, etc.), and the metadata associated with the organism from Battelle’s SoC database (whether or not the organism is a human pathogen, whether or not the organism can cause meningitis or respiratory disease, the types of respiratory disease, the likelihood that the organism causes disease, whether or not the organism is a common biological or environmental contaminant, references to substantiate the metadata, and other information). These results and metadata are used in the MDRE service (described above) for each disease type studied here (meningitis/encephalitis and respiratory disease), which are summarized in the “trigger-summary” tabs. Virulence and antimicrobial resistance (AMR) factors (both sample wide and agent specific) are described in downstream tabs, as detailed in File S1A.

### Data sets used for analysis.

Several data sets were used during this study, as described below.

**(i) *In silico* data set.** The complete reference genomes of 21 different organisms across the tree of life were downloaded from the NCBI RefSeq database using the open-source bioinformatic tool ncbi-genome-download v0.3.1 (https://github.com/kblin/ncbi-genome-download) with the commands described in its user manual. The downloaded genomes were combined, and a few sequences from the human genome were added to it to test the host removal process. The combined genomes were used to computationally generate a metagenomic sample using the open-source tool InSilicoSeq v1.5.4 (36) based on the Illumina HiSeq error model.

**(ii) Mock microbial community data sets.** To further test UltraSEQ’s taxonomic profiling capability, we analyzed data sets derived from mixed microbial communities as reported previously by Nicholls et al. (37). Specifically, the data sets under SRA accession numbers ERR3152364 (Nanopore data set) and ERR2984773 (Illumina data set) were run.

**(iii) Clinical data sets.** Clinical data sets are summarized in Table 3. The data sets represent a range of disease types, sample types, and sequence types. The final data sets (saliva respiratory data sets) were produced as part of this study, as described below (IRB no. 0782-100123699, Battelle Memorial Institute COVID-19 Biorepository).

**TABLE 3:** Clinical data sets analyzed in this study^*a*^

Data source or BioProject accession no. (study authors [reference{s}])	Disease type(s)	Sample type(s)	Data set type(s)	Clinical data provided by the study to compare to MDRE service results	Pipeline(s) compared to UltraSEQ
https://veb.lumc.nl/CliniMG (de Vries et al. [24])	Encephalitis and respiratory	CSF, brain, NP, BAL fluid, nasal wash, plasma	DNA and RNA reads	qPCR-positive patient samples (*n* = 13)	9 pipelines from reference 24
PRJNA516289 (Miller et al. [10])^*b*^	Encephalitis/meningitis	CSF	Illumina RNA and DNA reads	Original clinical tests (*n* = 216 tests) from 95 patient samples^*c*^	SURPI
PRJNA516582 (Saha et al. [12])	Encephalitis/meningitis	CSF	Illumina RNA reads	Clinical culture-, antigen-, or PCR-positive specimens (*n* = 36) from patients with neurologic infections based on negative specimens (*n* = 30) from patients with an alternate diagnosis; CHIKV PCR-positive specimens (*n* = 17); confirmed cases from an idiopathic set (*n* = 11)^*c*^	IDseq with logistic regression model
CSF_metagenomics from idseq.net (Hasan et al. [13])	Encephalitis/meningitis	CSF	Illumina DNA reads	Culture- and/or PCR-positive residual specimens (*n* = 74) from patients with suspected CNS infections^*d*^	IDseq
PRJEB7888 (Fischer et al. [15])	Respiratory	BAL fluid, sputum, nasal swab	Illumina RNA reads	Influenza PCR-positive respiratory specimens (*n* = 24) from patients with seasonal influenza infection and samples (*n* = 5) from patients with pneumonia that tested negative for influenza	Fischer et al.’s pipeline and Explify
PRJNA554856 (Watts et al. [16])	Respiratory	Endotracheal exudate	IonTorrent DNA reads	Culture-positive patient samples (*n* = 2)	Centrifuge and resistance gene identifier
PRJNA554461 (Yang et al. [18])	Respiratory	Endotracheal exudate	MinION DNA reads	Samples from patients (*n* = 14) with VAP, including culture positive (*n* = 9), culture negative (*n* = 5), and controls (*n* = 8) (patients without pneumonia)	WIMP
PRJEB13360 (Flygare et al. [19] and Graf et al. [20])	Respiratory	Pediatric NP/OP	Illumina RNA reads	PCR-positive samples (*n* = 24); no negative controls	Explify
PRJNA634356 (Babiker et al. [22])	Respiratory	NP	Illumina RNA reads	RT-PCR-negative (*n* = 30) and SARS-CoV-2 RT-PCR- and other PCR-positive patients with coinfections (*n* = 45)	Explify and KrakenUniq
Battelle (this study)	Respiratory	Saliva	Illumina RNA reads	SARS-CoV-2 RT-PCR-positive patients (*n* = 8)	NA

a CSF, cerebral spinal fluid; BAL, bronchial lavage; VAP, ventilator-associated pneumonia; NP, nasopharyngeal; OP, oropharyngeal; NA, not applicable; CHIKV, chikungunya virus; CNS, central nervous system.

b Note that for this case study, 89 samples were run for comparison to the data in Fig. 1B in reference 10. RNAseq data sets were run for RNA viruses, and DNAseq data sets were run for the rest. The 20 “challenge” samples (see Table S3 in the supplemental material in reference 10) were not included since the sample identifiers could not be matched to the raw data files. See “All Organisms” in Table S2 in the supplemental material in reference 10 (“MNA” identifications match up to the raw data sample identifications).

c Note that an additional 24 idiopathic meningitis cases (*n* = 24) were reported by those authors (12), but only those with confirmed tests were included in this study.

d Note that reads could be downloaded only with host reads subtracted in FASTQ format (raw files were not available). Furthermore, only 20 samples were available. Since most of these are true negatives, 5 samples with confirmed truth tests were selected for a proof-of-concept comparison to IDseq results.

### Processing of saliva samples for the Battelle data set.

Approximately 1- to 5-mL specimens were self-collected in RNase-free 50-mL Falcon tubes. Briefly, subjects were instructed to swallow a couple of times prior to the collection of normal saliva that naturally pools in the mouth without coughing or sniffing. After collection, the sample tubes were screwed shut and sterilized with a disinfecting alcohol wipe. Samples were submitted to a collection site within 30 min of collection, where they were immediately placed on ice. Samples were shipped to the laboratory and stored at 2°C to 8°C until diagnostic testing could be conducted. All samples were tested within 72 h of collection, and leftover saliva was stored at −70°C or lower for subjects who enrolled in the Battelle Biorepository.

### SARS-CoV-2 qPCR for Battelle samples.

Self-collected saliva samples were analyzed the same day using SalivaDirect DualPlex RT-qPCR targeting the N1 gene of SARS-CoV-2 and human RNase P. Briefly, samples were vortexed until they were homogeneous, and 50 μL of saliva was then mixed with 2.5 μL of MagMAX viral/pathogen proteinase K (Thermo Fisher) and vortexed at room temperature at 3,000 to 5,000 rpm for 1 min. Proteinase K was then heat inactivated for 5 min at 95°C prior to RT-PCR. RT-PCR was conducted using TaqPath one-step RT-qPCR master mix according to the SalivaDirect protocol on 5 μL of extraction-free saliva lysate. For each 96-well sample plate tested, a negative template control (NTC) and two positive controls (Twist synthetic SARS-CoV-2 RNA controls at 100 copies/μL) were assessed. Test results were valid only when the NTC returned a negative result and the positive controls returned the expected positive results for SARS-CoV-2 N1 and negative results for the RNase P gene. The detection criteria for clinical samples were as follows: if the SARS-CoV-2 N1 gene had a *C_T_* value of <37 and the RNase P gene had any value; it was a positive reportable result; if the RNase P gene had a *C_T_* value of <35 and the SARS-CoV-2 N1 gene had a *C_T_* value of ≥40, it was a reportable negative result; and if the RNase P gene had a *C_T_* value of ≥35 and the SARS-CoV-2 N1 gene had a *C_T_* value of ≥40, the test was considered invalid, and the test was repeated for that sample.

### Metatranscriptome sequencing for Battelle samples.

Saliva samples from each enrolled subject that returned a positive SARS-CoV-2 qPCR result underwent RNA extraction using the QIAamp viral RNA kit for QIAcube (Qiagen) according to the manufacturer’s instructions. RNA extracts were then treated using Kapa Hyper prep with RiboErase according to the manufacturer’s instructions. Library quantification was conducted using a Kapa library quant kit (Illumina) with universal qPCR mix (catalog number KK4824), and the libraries were normalized to 4 nM and pooled for sequencing with the Illumina NextSeq 500/550 high-output kit v2.5 (300 cycles, 150 by 150 bp). FASTQ data were processed through UltraSEQ as described above.

### Positive predictive agreement, negative predictive agreement, and accuracy.

The agreement of the metagenomic results with the pipeline was compared to reported molecular results (culture, qPCR, and antigen testing, etc.) in terms of positive predictive agreement (PPA) [PPA = TP/(TP + FN)], negative predictive agreement (NPA) [NPA = TN/(TN + FP)], and accuracy (ACC) [ACC = (TP + TN)/(TP + FN + FP + FN)]. In cases where negative samples were not available (e.g., de Vries et al.’s data set [24]), an additional metric, the positive predictive value (PPV), was used for comparisons, where PPV = TP/(TP + FP). Specifically, TPs are defined as instances where the bioinformatic pipeline identifies the same pathogen species as that reported by the molecular test, FNs are defined as instances where the molecular test identifies a pathogen but the bioinformatic pipeline does not, FPs are defined as instances where the bioinformatic pipeline identified a pathogen that was tested for but not identified by the molecular test, and TNs are defined as instances where the bioinformatic pipeline does not identify a pathogen and the molecular test also does not identify the pathogen. If no truth molecular result was available, the bioinformatic pipeline result was not scored.

For each published data set and corresponding pipeline, positive and negative calls were documented as reported in the publications, and the detailed results are described in File S3. In addition to the pipelines described in the studies in Table 3, for some respiratory data sets, the results were compared to Explify results using the Basespace app (21). For UltraSEQ, positive calls were made if an organism was identified in bin 1A, 1B, 2, or 3 according to the logic rules table described in File S1E, with the following exceptions. For all data sets, pathogens that rarely cause disease as annotated in Battelle’s SoC database were reported in the raw results but were not considered positive detections. For the data sets under BioProject accession numbers PRJNA554461, PRJNA516289, and PRJNA516582, additional filters were applied due to obvious E. coli contamination. Specifically, for the data set under BioProject accession number PRJNA554461, E. coli was considered a positive detection only if it was identified with a >5% relative abundance and >7 SoCs were identified; for the data set under BioProject accession number PRJNA516582, filters of a >50% relative abundance and >21 SoCs were applied; and for the data set under BioProject accession number PRJNA516289, E. coli-positive results were not reported under any condition.

### Data availability.

Metagenomic reads for the saliva samples from this study have been submitted to the NCBI BioProject database (https://www.ncbi.nlm.nih.gov/bioproject) under accession number PRJNA856680. The UltraSEQ platform is available for trial account use by contacting the authors.
