# Supplementary material for: UltraSEQ, a Universal Bioinformatic Platform for Information-Based Clinical Metagenomics and Beyond
Source: Microbiol Spectr. 2023 Apr 11;11(3):e04160-22. doi: 10.1128/spectrum.04160-22 (PMC10269449; doi:10.1128/spectrum.04160-22)
Supplement: Supplemental file 1 — Supplemental material. Download spectrum.04160-22-s0001.pdf, PDF file, 0.9 MB [file spectrum.04160-22-s0001.pdf]

Supplementary Materials

This manuscript contains the following supplemental materials:

Supplement A – Detailed UltraSEQ Services (this document)

Supplement B – Other UltraSEQ Services (this document)

Supplement C – Sample-report_user guide (separate Excel Document)

Supplement D – Supplemental_File_Scores (separate Excel Document)

Supplement E – UltraSEQ Rules Engine Logic (this document)

Supplement F – Supplemental Results (this document)

Supplement A – Detailed UltraSEQ Services

Preprocessing Service. For datasets derived from sequencers, UltraSEQ's preprocessing routine includes steps to trim low quality sequence regions, remove adapter sequences, (optionally) merge paired end reads, and (optionally) remove host sequences to ensure optimal reads remain for analysis. For Illumina and IonTorrent datasets, Trimmomatic v0.39 (Bolger et al., 2014) was used for quality trimming/adapter removal (settings include ILLUMINACLIP:NexteraPE-PE.fa:2:30:10:2:keepBothReads, LEADING:3 TRAILING:3 SLIDINGWINDOW:5:20 MINLEN:50) and Fastp v0.23.0 (Chen et al., 2018) was used for paired end read merging and deduplication (settings include -m (R1/R2 merging mode) when applicable, –dedup, -Q -A (disable quality and adapter trimming). *[Note: subsequent to this publication, UltraSEQ preprocessing routine was updated to use Fastp for quality trimming/adapter removal and merging in one step with the following setting: -m (R1/R2 merging mode), –cut_front –cut_front_window_size1 –cut_front_mean_quality 3 (mimics Trimmomatic LEADING:3), –cut_tail –cut_tail_window_size_1 –cut_tail_mean_quality 3 (mimics Trimmomatic TRAILING: 3), –cut_right –cut_right_window_size_5 –cut_right_mean_quality 20 (mimics Trimmomatic SLIDINGWINDOW:4:20), -l 50. A second step was used to deduplicate the dataset if necessary due to interference of Fastp's –cut_right setting on –dedup. We found that this pipeline provides 1.5x speed increase, ~1.07x more usable reads, and automatic adapter removal (data not shown)].* Following trimming and adapter removal, Bowtie2 v2.3.5.1 (Langmead & Salzberg, 2012) was used with default settings to remove host reads that produced an alignment to the human genome build GRCh38. For Nanopore datasets, Porechop v0.2.4 (Wick et al., 2017) was used with default settings to remove adapters and MiniMap2 v2.24-r1122 (Li, 2018) was used with default settings to remove any reads that produced an alignment to human genome build GRCh38. FastQC v0.11.9 (Andrews, 2010) and MultiQC v1.10.1 (Ewels et al., 2016) were used to evaluate pre-processed and post-processed standard data quality metrics and ensure preprocessing routines were effective.

To ensure the most informative reads are passed to the next UltraSEQ service, an additional de-duplication step is performed by removing any duplicates that have an exact match for the first 50 bases. Such duplication is known to occur during the library preparation step (Head et al., 2014). Further, to reduce cloud compute costs, enhance run-times, and provide better comparisons across datasets, subsampling was optionally performed prior to the alignment service described below. Subsampling was performed by calculating the average number of bps per read in a sample, then randomly sampling to the number of reads required to reach 10,100,000 total bps (note: the subsampling was performed by bps instead of number of reads since some datasets, e.g., nanopore, have much longer reads). Subsampling was performed on all datasets (if needed) with the exception of a second set of runs that was performed the for Yang et al. (Yang et al., 2019) datasets for antibiotic resistance genotyping. For these runs, a separate UltraSEQ run was used in which the full sample was run without de-hosting to enhance signals for antibiotic resistance genes (with the exception of the following samples that were subsampled to 30,000 reads: Case 22, 10, 9, and 4; these were subsampled to 30,000 reads to reduce computational runtime).

Aligner Service. To avoid sequences with lengths longer than LAMBDA2's maximum query length, sequences longer than 5,000 bps were chunked in pieces of maximum size 5,000.

LAMBDA2 enables alignment against both protein and nucleotide databases, but the results for this study leveraged only protein databases, including the Uniref100 protein database (built April 2021) and Battelle's Sequence of Concern protein database, which contains ~8,000 sequences of concern (including virulence factors, toxins, bioregulators, pathways of concern, etc.), ~500 signatures of genetic engineering, and ~3,500 biological agents, including ~2,800 pathogens (~2,600 human pathogens) as detailed here (Gemler et al., 2022). For selected runs, we also leveraged our curated nucleotide database of human pathogens and select agents, although these data are not shown here, as no improvement to results was noticed. For this study, the following aligner settings were used: e-value = 1e-4, maximum number matches = 10, aligner's seed-delta-increases-length flag = ON.

Query Mapper Service: This service maps regions within query sequences to identify high quality alignment regions as well as chimeric reads / out-of-context DNA sequences. This service processes the raw alignment results from the aligner service and identifies top alignment results by first finding the top percent identity and then subsetting the raw alignment results to alignments whose percent identity is within a tolerance, by default 1%, of the top percent identity. The top alignment results are subsequently processed for positional information from each database used, including protein and nucleotide databases. For each query position, n_{counts} is defined as the total number of query alignment starts and query alignment stops corresponding to that position. After n_{counts} has been populated at every position, a normalized vector of counts (N_{counts}) is compiled according to equation 1:

Equation 1

$$N_{counts} = \frac{n_{counts} - Min(n_{counts})}{Max(n_{counts}) - Min(n_{counts})}$$

Following these calculations, a K-means clustering is performed for the N_{counts} values, and the top cluster is used to define the region bounds. Specifically, kmeans++ (Arthur & Vassilvitskii, 2007) is used to set the initial centroids. For this application, the "furthest point" algorithm sequentially selects initial centroids furthest from the ones in the previous iteration. Lloyd's Algorithm (Lloyd, 1982) is then used for clustering given those initial centroids. Finally, the Elbow Method (Ng, 2012) is used to determine the best number of groups k. The top cluster is defined as the cluster with the largest centroid. Further, as implemented within UltraSEQ, the algorithm checks if the top two clusters' centroids are within a particular tolerance (10% in the case of the query mapper); if they are, the penultimate cluster is absorbed into the top cluster (otherwise, the top cluster remains unaltered).

To illustrate these calculations, consider the following example: one query sequence of length 150 bps aligns to 3 different subject sequences, with the following start and stop query positions (and percent identities): Accession A, start position 1, end position 100 (percent identity = 100); Accession B, start position 1, end position 50 (percent identity = 95); Accession C, start position 25, end position 100 (percent identity = 100). In this case, n_{counts} = 195, 100, 95, and 200 and N_{counts} = 0.952, 0.0476, 0, 1.000, for positions 1, 25, 50, and 100, respectively. In this case, the top K-means cluster includes N_{counts} values 0.95 and 1.000 associated with query positions 1 and 100, respectively. Query positions 1 and 100 then define the region bounds. The region

bounds are subsequently applied to the query sequence, and any overhangs of sufficient length (default: 6 bps) can optionally be classified as their own region (overhangs that are less than the sufficient length threshold are ignored). The default setting for this study was to generate overhangs when possible. The query mapper also defines the region's type: if the region has one or more alignments derived from a protein database, it is defined as a “translated” region; if it only has alignments from a nucleotide database, it is defined as an “untranslated” region; if no alignments are identified, it is defined as a “novel” region. Further, for translated regions, the reading frame(s) is documented based on the alignment. UltraSEQ provides the option to re-align novel regions for greater depth of analysis, but this option was not used in this study. In the example presented here, two regions would be identified: one from query positions 1 to 100, and the second from query positions 101 to 150.

Context Services and Subservices. These services generate contextual information and passes information to downstream services. The **Metadata Service** maps metadata to alignment results. For UniRef100 alignments, these metadata include Gene Ontology terms, UniProt identifiers, UniRef100 identifiers (which are linked to proteins involved in genetic engineering, housed within Battelle's SoC database), taxonomy identifiers (also linked to Battelle's SoC database for agent metadata), and other. For SoC alignments, these metadata further include tags such as coarse functionality (adherence, antibiotic resistance, etc.), pathways, SoC groups, etc. as defined in (Gemler et al., 2022). For nucleotide alignments, current metadata includes taxonomic identifiers. Other context services available for use but not used in this study are described in the Supplement B - Other UltraSEQ Services.

Rules Engine Service. This service combines all of the above context and prediction services for regions, sequences, and samples using user defined logic rules for rapid sequence triage. UltraSEQ currently has 4 default rules engines to identify biothreats, controlled sequences for DNA synthesis vendors, indicators of genetic engineering, and Metagenomics Diagnostics. The first three are not described here as they are specific to various use cases. The fourth is described in the main methods of the manuscript.

Metagenomics Service. This service provides sample level taxonomic composition based on the regions identified from reads processed in the query mapper service in 3 steps: 1) filtering out low quality reads, 2) scoring the remaining reads based on the information content of the reads, and 3) predicting the taxonomic composition based on the scores. In the first step, the default alignment quality filters used in this study include minimum alignment length of 48 base pairs (16 amino acids set based on aligner seed length), 99% percent identity and 100% region coverage for nucleotide alignments (note: no nucleotide databases were used in this study), 95% percent identity and 90% region coverage for protein alignments.

The metagenomics service works by estimating the information content of a read. That is, reads that are unique to a protein from a specific organism contain the highest amount of information, whereas reads that are found in proteins from across the tree of life contain less information. The information content of a read is derived from the read's alignment data, in which the value of its information content is inversely proportional to the product of the number of unique accessions and taxonomies associated with high-quality alignments of a region – i.e., a region that contains a single accession and taxonomy call is more useful than a region that contains

many accession and taxonomy calls. We note that the default protein reference database used in this study, the UniRef100, clusters proteins with 100% similarity to each other into a single reference accession. This clustering feature is important for the metagenomics service's efficacy, since it prevents reference database duplication from incorrectly lowering the perceived information content of a region (e.g., duplicates of the same protein are represented by a single UniRef100 cluster, which would appear as a single subject accession in this study).

Sequence region-level taxonomy predictions are associated with confidence scores that are based on alignment quality. For each unique taxonomy identified, the maximum confidence score from alignments that are associated with it are assigned. Specifically, based on the results of the query mapper service, all region alignments are compiled in a table ("query sequence information table"), and scoring is initially performed on a per region, per agent (organism), per accession basis. More specifically, each region (r), agent (a), and accession (acc) combination is assigned the following score, $S_{a,r,acc}$:

$$S_{a,r,acc} = \frac{Aqual_{acc,r}}{Na_r \times Nacc_r}$$

Where $Aqual_{acc,r}$ is the alignment quality (percent coverage x percent identity) in the region, Na_r is the number of unique agents associated with the subject accessions in the region (score is inversely proportional to the region's uniqueness) and $Nacc_r$ is the number of accessions from the subject database that are associated with the region (score is inversely proportional to the region's sequence complexity – higher complexity implies more specificity to a specific protein). Subsequently, an agent region score, $S_{a,r}$, is calculated to be the score associated with the highest scoring accession (or accessions in the case of a tie) for the given agent, region combination:

$$S_{a,r} = \max_{acc} S_{a,r,acc}$$

For each unique taxonomy across the sample, the agent scores $S_{a,r}$ are summed across all regions for which each taxonomy is associated, and the sample-level or agent score (S_a) is calculated:

$$S_a = \sum_r S_{a,r}$$

At this point, the agent score (S_a) are rank ordered, and starting with highest sample-level scoring taxonomy, all sequences associated with the highest scoring taxonomy are identified and all other taxonomies associated with those sequences are removed from the query sequence information table (as defined above). This process is iteratively repeated until all taxonomies have been processed. The result is a pruned list of agent scores (S_a) and their associated TaxIDs. From this list, a K-means cluster of the agent scores is performed by domain (Bacteria, Archaea, Eukaryotes, and Viruses) using the same method as described above for the Query Mapper Service, and the taxonomies associated with the top cluster in each domain are set to be the final sample composition. As with the query mapper, the algorithm checks if the top two clusters' centroids are within a particular tolerance, referred to as

the metagenomic clustering threshold (MCT) in the main body of the manuscript; if they are, the penultimate cluster is absorbed into the top cluster (otherwise, the top cluster remains unaltered). In this case, a 50% MCT was used for all UltraSEQ runs except during testing phases as described in the Results Section. The final confidence associated with each agent, C_a, is defined as the average alignment quality for all sequences used in the final (pruned) query sequence information table for that agent. Note, due to the high abundance of phages, all TaxIDs associated with phages and other similar non-human viruses were masked from these calculations. This masking was accomplished by creating a removal list of all NCBI viral TaxID associated with the following hosts: fungi, bacteria, algae, archaea, diatom, and protozoa.

Reporting Services. UltraSEQ provides several reports as well as described below. The text below describes the details of these reports as of the writing of this manuscript, although we anticipate additions/modifications as appropriate. Details for the *Top Alignment Repor*t, *Taxonomy Report,* and *Default Report* are provided in the main section of the manuscript. Additional details for the sample report are provided here.

***Sample Report*.** As described in the main methods section of the manuscript, the ‘main report’ tab provides a list of all organisms identified from the above Metagenomics Service, the results associated with the identified organisms, and the metadata associated with the organism from Battelle’s SoC database. These results and metadata are used in a logical diagnostic rules engine described in the ‘trigger-summary’ tabs. Statistics for the UltraSEQ run are provided in the ‘sample-statistics’ tab. For each organism identified, the ‘VF’ tab provides a list of SoCs identified, including virulence factors and antibiotic resistance genes from the Comprehension Antibiotic Resistance Database (CARD) (Alcock et al., 2020). Specifically, if an alignment to one of the proteins in the SoC database is contained within the Top Alignment Report and its TaxID matches to the organism or one of its children, it is populated in the organism-specific ‘VF’ tab. For antibiotic resistance profiles, only proteins in the CARD’s protein homology model are currently used. These protein sequences are currently populated in Battelle’s SoC database, but some metadata associated with these sequences and the drugs they confer resistance to defined by CARD (e.g., in the aro.tsv and ro.tsv files provided by CARD downloads https://card.mcmaster.ca/download). The ‘ABR’ tab pulls results from the ‘VF’ tab to provide antibiotic resistance information. Information in this tab is organized by drug class and antibiotic for easy interpretation. Other antibiotic resistance models (e.g., protein variant model, rRNA gene variant model, and protein knockout model, etc.) are not currently used in UltraSEQ. Thus, antibiotic resistance profiles are currently based solely on presence of genes that confer antibiotic resistance (i.e., profiles are not based on point mutants that may help confer resistance). In addition to the organism-specific antibiotic resistance profile, an organism-specific agnostic profile is provided in the ‘CARD SoCs Report’ tab to further aid in antibiotic resistance genotyping (in cases where antibiotic genes may map to the incorrect or many different taxonomies).

References for Supplemental Section A

1. Alcock, B. P., Raphenya, A. R., Lau, T. T. Y., Tsang, K. K., Bouchard, M., Edalatmand, A., Huynh, W., Nguyen, A. V., Cheng, A. A., Liu, S., Min, S. Y., Miroshnichenko, A., Tran, H. K., Werfalli, R. E., Nasir, J. A., Oloni, M., Speicher, D. J., Florescu, A., Singh, B., . . . McArthur, A. G. (2020). CARD 2020: antibiotic resistome surveillance with the comprehensive antibiotic resistance database. *Nucleic Acids Res*, *48*(D1), D517-D525. https://doi.org/10.1093/nar/gkz935
2. Andrews, S. (2010). *FastQC: A Quality Control Tool for High Throughput Sequence Data [Online]. Available online at: http://www.bioinformatics.babraham.ac.uk/projects/fastqc/*.
3. Arthur, D., & Vassilvitskii, S. (2007). K-means++: the advantages of careful seeding. SODA'07 Proceedings of the Eighteenth Annual ACM-SIAM Symposium on Discrete Algorithms. CityPhiladelphia, StatePA: placecountry. In: region SIAM Press.
4. Bolger, A. M., Lohse, M., & Usadel, B. (2014). Trimmomatic: a flexible trimmer for Illumina sequence data. *Bioinformatics*, *30*(15), 2114-2120. https://doi.org/10.1093/bioinformatics/btu170
5. Chen, S., Zhou, Y., Chen, Y., & Gu, J. (2018). fastp: an ultra-fast all-in-one FASTQ preprocessor. *Bioinformatics*, *34*(17), i884-i890. https://doi.org/10.1093/bioinformatics/bty560
6. Ewels, P., Magnusson, M., Lundin, S., & Kaller, M. (2016). MultiQC: summarize analysis results for multiple tools and samples in a single report. *Bioinformatics*, *32*(19), 3047-3048. https://doi.org/10.1093/bioinformatics/btw354
7. Gemler, B. T., C., M., A., H. C., D., H., Z., S., J., H. L., O., T., & C., B. (2022). Function-based Classification of Hazardous Biological Sequences: Demonstration of a New Paradigm for Biohazard Assessments (Submitted for Publication).
8. Head, S. R., Komori, H. K., LaMere, S. A., Whisenant, T., Van Nieuwerburgh, F., Salomon, D. R., & Ordoukhanian, P. (2014). Library construction for next-generation sequencing: overviews and challenges. *Biotechniques*, *56*(2), 61-64, 66, 68, passim. https://doi.org/10.2144/000114133
9. Langmead, B., & Salzberg, S. L. (2012). Fast gapped-read alignment with Bowtie 2. *Nat Methods*, *9*(4), 357-359. https://doi.org/10.1038/nmeth.1923
10. Li, H. (2018). Minimap2: pairwise alignment for nucleotide sequences. *Bioinformatics*, *34*(18), 3094-3100. https://doi.org/10.1093/bioinformatics/bty191
11. Lloyd, S. (1982). Least squares quantization in PCM. *IEEE transactions on information theory*, *28*(2), 129-137.
12. Ng, A. (2012). Clustering with the k-means algorithm. *Machine Learning*, 1-2.
13. Wick, R. R., Judd, L. M., Gorrie, C. L., & Holt, K. E. (2017). Completing bacterial genome assemblies with multiplex MinION sequencing. *Microb Genom*, *3*(10), e000132. https://doi.org/10.1099/mgen.0.000132
14. Yang, L., Haidar, G., Zia, H., Nettles, R., Qin, S., Wang, X., Shah, F., Rapport, S. F., Charalampous, T., Methe, B., Fitch, A., Morris, A., McVerry, B. J., O'Grady, J., & Kitsios, G. D. (2019). Metagenomic identification of severe pneumonia pathogens in mechanically-ventilated patients: a feasibility and clinical validity study. *Respir Res*, *20*(1), 265. https://doi.org/10.1186/s12931-019-1218-4

Supplement B: Other UltraSEQ Services

Other Context Services and Subservices. Other services include a **Genetic Engineering (GE) Service** and a **Classifier Service.** The **GE Service enables** prediction of GE indicators, including microservices for detection of GE proteins, GE signatures, codon optimization, and codon re-coding. The Classifier Service includes artificial intelligence (AI) models to make alignment-free predictions on amino acid sequences; the output is the probability that the input is associated with a subset of threat metadata categories (the coarse functional categories) described in Gemler et al. (Gemler et al., 2022). Information from the context services are passed to the Prediction Services and Flagging System (Rules Engine) as described below.

Region-based Taxonomy Prediction Subservice. For other applications (such as forensic applications), sequence and region-level taxonomic information can be useful. For this prediction, a conservative and information-based approach is used that takes into consideration strength of alignments, the number of times a TaxID appears across alignments, and the taxonomic depth (species, genus, etc.). For this prediction, the TaxID frequency that each taxID appears across the top alignments is calculated, and TaxID Depth is assigned as follows: 100 for species and below, 75 for genus, 50 for family, 30 for order, 20 for class, 15 for phylum, and 10 for domain and above. A normalized TaxID Depth is then calculated in the same manner as the normalization defined in Equation 1 (**Supplemental Material A**). The TaxID score for each TaxID is then calculated according to Eq 2.

Equation 2

$$TaxID\ Score = \sqrt{w_d\,(Normalized\ TaxID\ Depth)^2 + w_f(TaxID\ Frequency)^2}$$

Where default weight values: w_d = 2.0 and w_f = 1.0 are used (optimized weights based on test/validation datasets, not shown). The final taxonomy predictions are then based on a 2-D K-means clustering for the alignment confidence and TaxID score data. The TaxIDs in the top cluster are considered the final predictions using the same K-means clustering methods as described above (the rest of the TaxID predictions are discarded. Further, the confidence associated with each TaxID prediction is reported as follows: taxonomy evidence is gathered for the region from the alignments and the alignment scores (percent identity x percent region coverage) are normalized by the max heuristic value (100 in the case of 100% identity over 100% of the region). For each TaxID identified, this normalized score is considered the final confidence score associated with each TaxID.

Region-based Function Prediction Subservice. For each region, the function (gene ontology terms) is calculated in a similar manner. Specifically, 1) function evidence is gathered for each Region and alignment scores are normalized, 2) a 1-D K-means clustering is used for the alignment confidence; the GO Term sets in the top cluster are selected, and 3) the final function prediction confidence is calculated by averaging the alignment confidence values across the members in the top cluster.

Region-based Threat Prediction Subservice. For each region, the threat metadata associated with that region (damage, antibiotic resistance, adherence, etc. as defined in

(Gemler et al., 2022)) is tabulating the SoC alignment scores (percent identity x region coverage) associated with threat metadata category, clustering the scores using K-means, down selecting to only the top cluster, adding the alignment scores to 1/100th of the AI model, then dividing by the maximum possible score.

References for Supplemental Section B

1. Gemler, B. T., C., M., A., H. C., D., H., Z., S., J., H. L., O., T., & C., B. (2022). Function-based Classification of Hazardous Biological Sequences: Demonstration of a New Paradigm for Biohazard Assessments (Submitted for Publication).

Supplement E - UltraSEQ Rules Engine Logic

Bin	Disease Diagnosis Confidence	Qualifications
Bin 1A	Highest Confidence Agent	1. SoC Filter* is True AND<br>2. SoC agent is a pathogen that infects human host AND<br>3. Relative abundance filters (based on predicted reads): 1%-5% for bacteria**; > 1%-2%** for fungi; >1% for other (protozoa, etc.) AND<br>4. SoC agent is contained in Battelle's human respiratory pathogen list (for respiratory datasets) or encephalitis/ meningitis pathogen list (for encephalitis datasets) AND***<br>5. At least one SoC with the Active, Damage, Apoptosis, Inhibits, or Transmission threat category from the agent was used by UltraSEQ's metagenomics module for agent prediction
Bin 1B	High Confidence Agent	1. SoC Filter* is False AND Conditions 2,3,4, and 5 above met
Bin 2	Medium Confidence Respiratory Agent	1. Condition 1A and Condition1B = FALSE AND Conditions 2 and 3 above met AND<br>2. No SoCs identified from the above categories for that agent
Bin 3	Lowest Confidence Respiratory Agent	1. Condition 1A and Condition1B = FALSE AND Conditions 2 and 3 above met AND<br>2. There are SoCs from the above categories for that agent, however none are found [Note: other SoCs may be identified such as antibiotic resistant SoCs and adherence SoCs]

* SoC Filter is a condition that is true when the UltraSEQ metagenomics service uses a UniRef100 cluster containing a SoC to trigger the taxonomy prediction.
** For bacteria, a 5% threshold was used for the de Vries et al., PRJNA516289, Hasan et al., PRJEB7888, PRJEB13360; a 1% bacteria filter was used for all other datasets; for fungi, a 2% filter was used PRJNA516582; a 1% fungi filter was used for all other datasets
*** At the time of this manuscript, Battelle's SoC database contained ~2,200 human pathogen species, ~150 of which are curated as potential contaminants (either from reagents used during sequencing and/or due to the biological sample such as normal skin flora; all of these annotations are provided in the sample report. Of the human pathogens, ~250 are contained within the encephalitis/ meningitis list and ~250 are contained within the respiratory list.

Supplement F - Supplemental Results

Encephalitis / meningitis

PRJNA516289 (Miller et al. (Miller et al., 2019)).

Table F1. UltraSEQ Results for Miller Dataset

Result	Parasites		Fungi		Bacteria		DNA Viruses		RNA Viruses	
	UltraSEQ	Miller	UltraSEQ	Miller	UltraSEQ	Miller	UltraSEQ	Miller	UltraSEQ	Miller
TP	1	1	10	9	6	5	23	25	10	11
FP	0	0	0	0	0	1	0	0	0	0
FN	0	0	0	1	1	2	5	3	3	2
TN	4	4	38	38	51	50	16	16	5	5
PPA	100%	100%	100%	90%	86%	71%	82%	89%	77%	85%
NPA	100%	100%	100%	100%	100%	98%	100%	100%	100%	100%
Accuracy	100%	100%	100%	98%	98%	95%	87%	93%	83%	89%

* As noted by Miller et al., the "truth" was considered the initial clinical result unless a confirmatory test was run (i.e., if a confirmatory test was run by Miller et al., the truth was considered to be the confirmatory test). For both SURPI and UltraSEQ, RNA viruses were reported using sequences derived from the RNA libraries, whereas all other organisms results were based on sequences from the DNA libraries.

PRJNA516582 (Saha et al. (Saha et al., 2019)).

Table F2: Summary of Results for Saha and UltraSEQ

Result	Saha Results				UltraSEQ Results			
	All samples	Culture Only	All confirmed Cases	CHIKV cases	All samples	Culture Only	All confirmed Cases	CHIKV cases
TP	52	7	24	17	53	7	25	17
FP	NR*	NR*	NR*	NR*	0	0	0	0
FN	12	1	12	0	11	1	11	0
TN	29	N/A	N/A	N/A	29	N/A	N/A	N/A
PPA	81%	88%	67%	100%	83%	88%	72%	100%
NPA	N/A	N/A	N/A	N/A	100%	N/A	N/A	N/A
ACC	87%	88%	67%	100%	88%	88%	72%	100%

* NR= not reported; N/A=not applicable (i.e., could not be calculated)

** As detailed in the methods, UltraSEQ identified *E. coli* in nearly every sample despite the fact that *E. coli* was only identified by clinical tests in 2 samples. By using the UltraSEQ logic as defined in the Methods section without any background sample subtraction (as required by Saha), UltraSEQ was able to remove all *E. coli* false positives.

'CSF_metagenomics' from idseq.net (Hasan et al. (Hasan et al., 2020)).

Table F3: *Table of Species Identified by UltraSEQ for Sample CW322*

Taxonomy Name	TaxID	NCBI TaxID Rank	Type	Confidence	Relative Abundance TaxID + Children (vs PREDICTED ONLY reads)
Neisseria meningitidis	487	species	Bacteria	99.4	99.96
Human alphaherpesvirus 2	10310	species	Virus	99.0	0.0025

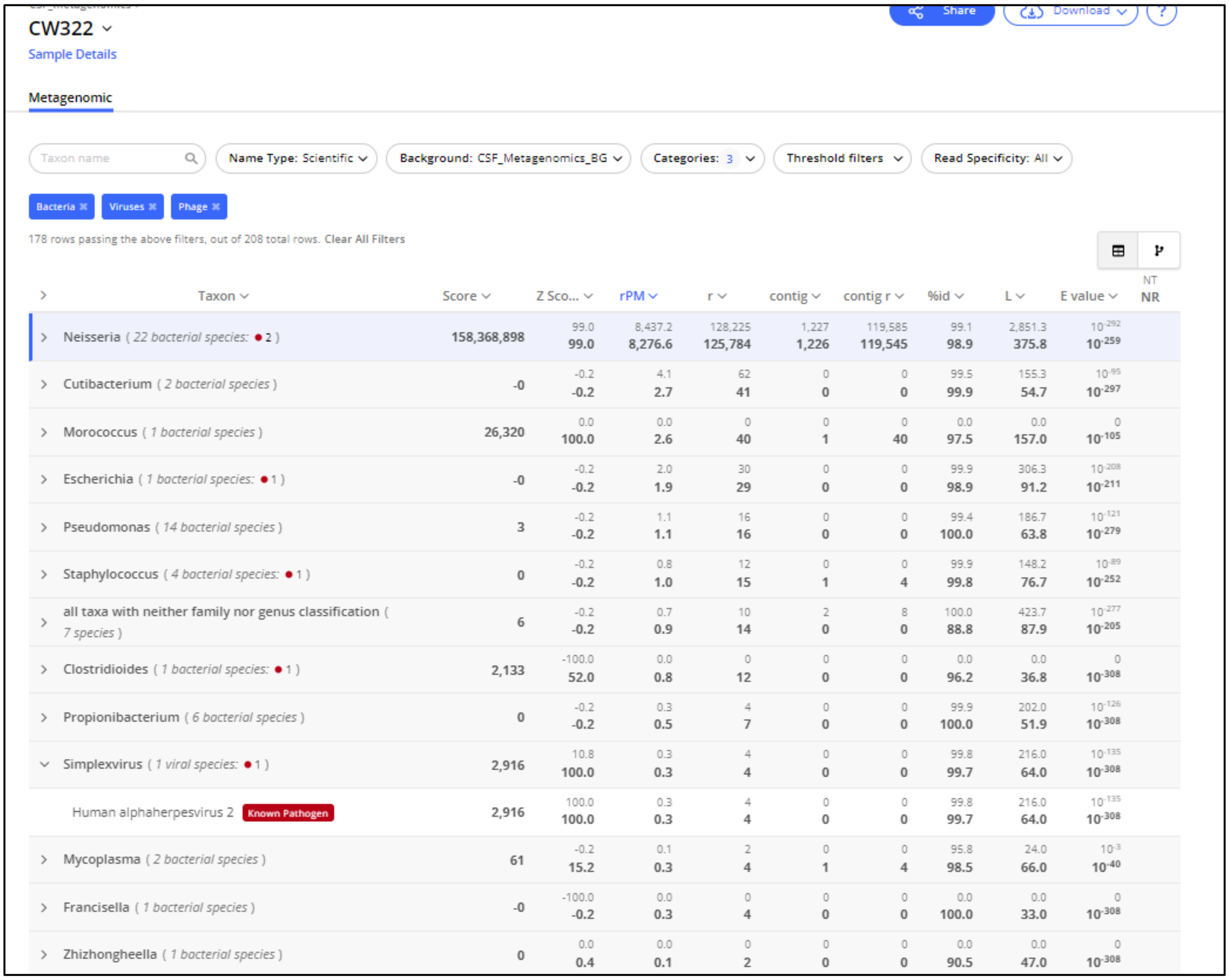

Taxon	Score	Z Sco...	rPM	r	contig	contig r	%id	L	E value	NT<br>NR
Neisseria (*22 bacterial species:* ● 2)	158,368,898	99.0<br>99.0	8,437.2<br>8,276.6	128,225<br>125,784	1,227<br>1,226	119,585<br>119,545	99.1<br>98.9	2,851.3<br>375.8	10^{-292}<br>10^{-259}	
Cutibacterium (*2 bacterial species*)	-0	-0.2<br>-0.2	4.1<br>2.7	62<br>41	0<br>0	0<br>0	99.5<br>99.9	155.3<br>54.7	10^{-95}<br>10^{-297}	
Morococcus (*1 bacterial species*)	26,320	0.0<br>100.0	0.0<br>2.6	0<br>40	0<br>1	0<br>40	0.0<br>97.5	0.0<br>157.0	0<br>10^{-105}	
Escherichia (*1 bacterial species:* ● 1)	-0	-0.2<br>-0.2	2.0<br>1.9	30<br>29	0<br>0	0<br>0	99.9<br>98.9	306.3<br>91.2	10^{-208}<br>10^{-211}	
Pseudomonas (*14 bacterial species*)	3	-0.2<br>-0.2	1.1<br>1.1	16<br>16	0<br>0	0<br>0	99.4<br>100.0	186.7<br>63.8	10^{-121}<br>10^{-279}	
Staphylococcus (*4 bacterial species:* ● 1)	0	-0.2<br>-0.2	0.8<br>1.0	12<br>15	0<br>1	0<br>4	99.9<br>99.8	148.2<br>76.7	10^{-89}<br>10^{-252}	
all taxa with neither family nor genus classification (*7 species*)	6	-0.2<br>-0.2	0.7<br>0.9	10<br>14	2<br>0	8<br>0	100.0<br>88.8	423.7<br>87.9	10^{-277}<br>10^{-205}	
Clostridioides (*1 bacterial species:* ● 1)	2,133	-100.0<br>52.0	0.0<br>0.8	0<br>12	0<br>0	0<br>0	0.0<br>96.2	0.0<br>36.8	0<br>10^{-308}	
Propionibacterium (*6 bacterial species*)	0	-0.2<br>-0.2	0.3<br>0.5	4<br>7	0<br>0	0<br>0	99.9<br>100.0	202.0<br>51.9	10^{-126}<br>10^{-308}	
Simplexvirus (*1 viral species:* ● 1)	2,916	10.8<br>100.0	0.3<br>0.3	4<br>4	0<br>0	0<br>0	99.8<br>99.7	216.0<br>64.0	10^{-135}<br>10^{-308}	
Human alphaherpesvirus 2 Known Pathogen	2,916	100.0<br>100.0	0.3<br>0.3	4<br>4	0<br>0	0<br>0	99.8<br>99.7	216.0<br>64.0	10^{-135}<br>10^{-308}	
Mycoplasma (*2 bacterial species*)	61	-0.2<br>15.2	0.1<br>0.3	2<br>4	0<br>1	0<br>4	95.8<br>98.5	24.0<br>66.0	10^{-3}<br>10^{-40}	
Francisella (*1 bacterial species*)	-0	-100.0<br>-0.2	0.0<br>0.3	0<br>4	0<br>0	0<br>0	0.0<br>100.0	0.0<br>33.0	0<br>10^{-308}	
Zhizhongheella (*1 bacterial species*)	0	0.0<br>0.4	0.0<br>0.1	0<br>2	0<br>0	0<br>0	0.0<br>90.5	0.0<br>47.0	0<br>10^{-308}	

Figure F1: *CW322 Results Showing Bacteria and Viruses Identified by IdSeq (Note that several more rows of genera were identified and not shown here).*

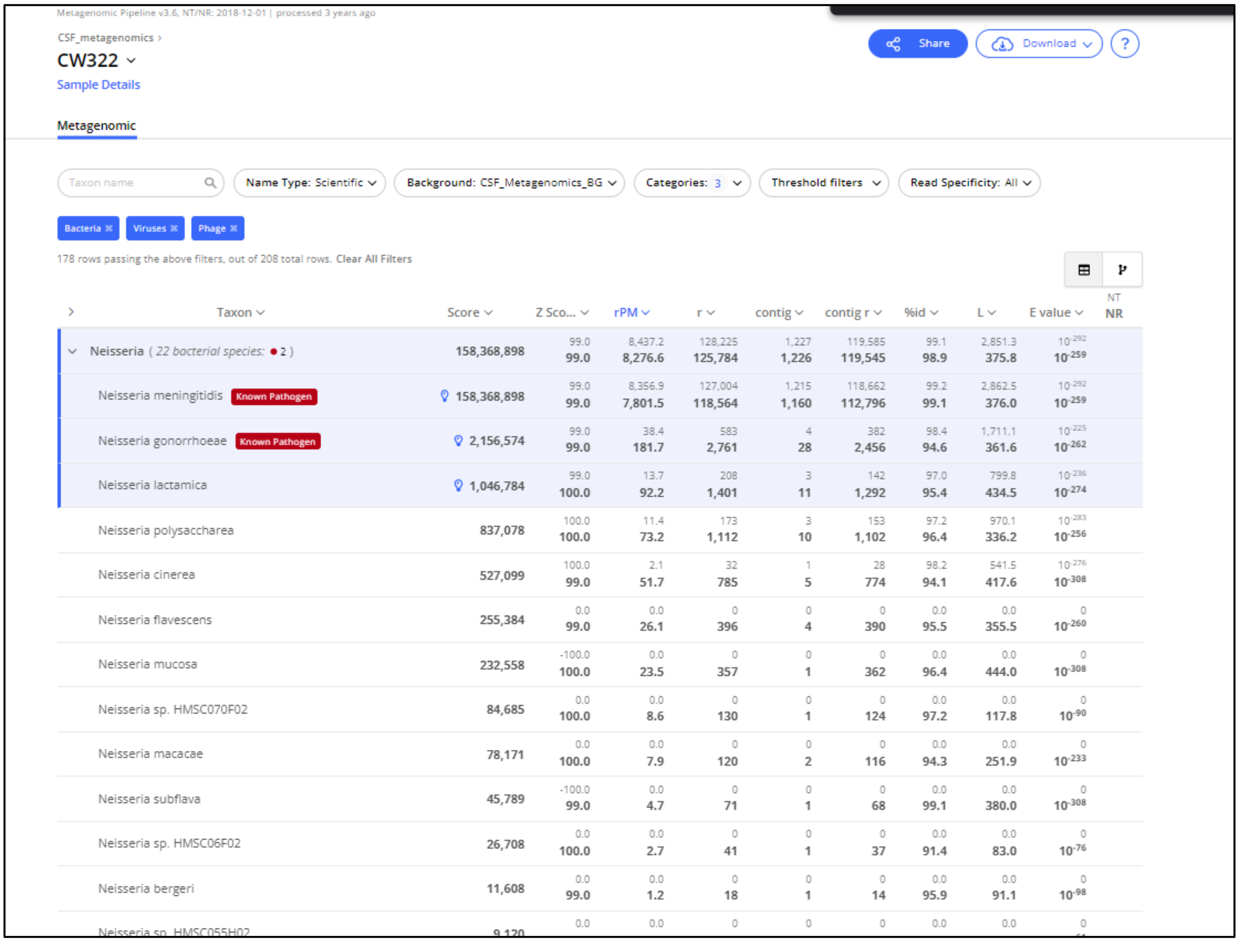

Figure F2: *CW322 Results Showing all Neisseria Species Identified (Note that several more rows of species were identified and not shown here).*

Respiratory disease: Influenza

PRJEB7888 (Fischer et al. (Fischer et al., 2015)).

Table F4: Summary of Results for Fischer and UltraSEQ

Pipeline	TP	FN	TN	FP	PPA	NPA	Accuracy
Explify	16	3	5	0	84%	100%	83%
Fisher	15	4	5	0	79%	100%	88%

UltraSEQ	14	5	5	0	74%	100%	79%

Respiratory disease: ventilator associated pneumonia (VAP)

PRJNA554856 (Watts et al. (Watts et al., 2019)).

Table F5: *AbR Report for SRR9693434 (Patient 2, Day 1)*

**fluoroquinolone antibiotic (ARO:0000001)**		[‘major facilitator superfamily (MFS) antibiotic efflux pump (ARO:0010002)’, ‘Staphylococcus aureus norA’, [‘ARO:3004667’], 31, 0.016, ‘0’] [‘major facilitator superfamily (MFS) antibiotic efflux pump (ARO:0010002)’, ‘Staphylococcus aureus norA’, [‘ARO:3004667’], 31, 0.016, ‘0’] [‘major facilitator superfamily (MFS) antibiotic efflux pump (ARO:0010002)’, ‘Staphylococcus aureus norA’, [‘ARO:3004667’], 31, 0.016, ‘0’] [‘major facilitator superfamily (MFS) antibiotic efflux pump (ARO:0010002)’, ‘Staphylococcus aureus norA’, [‘ARO:3004667’], 31, 0.016, ‘0’]	ciprofloxacin (ARO:0000036) enoxacin (ARO:0000023) ofloxacin (ARO:3000663) norfloxacin (ARO:3000662)	[‘Staphylococcus aureus norA’, [‘ARO:3004667’], 31, 0.016, ‘0’]	[‘major facilitator superfamily (MFS) antibiotic efflux pump (ARO:0010002)’, ‘Staphylococcus aureus norA’, [‘ARO:3004667’], 31, 0.016, ‘0’]
**phosphonic acid antibiotic (ARO:0000025)**		[‘fosfomycin thiol transferase (ARO:3000133)’, ‘FosD’, [‘ARO:3004674’], 1, 0.001, ‘0’]			

**glycylcycline (ARO:0000042)**	['mepA', ['ARO:3000026'], 44, 0.023, '0']	['multidrug and toxic compound extrusion (MATE) transporter (ARO:3000112)', 'mepA', ['ARO:3000026'], 44, 0.023, '0']	tigecycline (ARO:0000030)	['mepA', ['ARO:3000026'], 44, 0.023, '0']	['multidrug and toxic compound extrusion (MATE) transporter (ARO:3000112)', 'mepA', ['ARO:3000026'], 44, 0.023, '0']
**lincosamide antibiotic (ARO:0000017)**		['ABC-F ATP-binding cassette ribosomal protection protein (ARO:3004469)', 'poxtA', ['ARO:3004470'], 6, 0.003, '0'] ['ABC-F ATP-binding cassette ribosomal protection protein (ARO:3004469)', 'vgaA', ['ARO:3002829'], 1, 0.001, '0'] ['ABC-F ATP-binding cassette ribosomal protection protein (ARO:3004469)', 'vgaC', ['ARO:3002831'], 1, 0.001, '0']			
**macrolide antibiotic (ARO:0000000)**		['macrolide phosphotransferase (MPH) (ARO:3000333)', 'mphC', ['ARO:3000319'], 72, 0.037, '0'] ['ABC-F ATP-binding cassette ribosomal protection protein (ARO:3004469)', 'poxtA', ['ARO:3004470'], 6, 0.003, '0'] ['ABC-F ATP-binding cassette ribosomal protection protein (ARO:3004469)', 'vgaA', ['ARO:3002829'], 1, 0.001, '0'] ['ABC-F ATP-binding cassette ribosomal protection protein (ARO:3004469)', 'vgaC', ['ARO:3002831'], 1, 0.001, '0']	spiramycin (ARO:3000156) clarithromycin (ARO:0000065) roxithromycin (ARO:0000027) tylosin (ARO:3000145) oleandomycin (ARO:3000867) azithromycin (ARO:3000158) erythromycin (ARO:0000006) dirithromycin (ARO:3000176) telithromycin (ARO:0000057)	['mphC', ['ARO:3000319'], 72, 0.037, '0']	['macrolide phosphotransferase (MPH) (ARO:3000333)', 'mphC', ['ARO:3000319'], 72, 0.037, '0']
**mupirocin (ARO:3000554)**		['antibiotic-resistant isoleucyl-tRNA synthetase (ileS) (ARO:3000446)', 'mupA', ['ARO:3000521'], 10, 0.005, '1'] ['antibiotic-resistant isoleucyl-tRNA synthetase (ileS) (ARO:3000446)', 'mupB',	mupirocin (ARO:3000554)	['mupA', ['ARO:3000521'], 10, 0.005, '1'] ['mupB', ['ARO:3000510'], 2, 0.001, '1']	['antibiotic-resistant isoleucyl-tRNA synthetase (ileS) (ARO:3000446)', 'mupA', ['ARO:3000521'], 10, 0.005, '1'] ['antibiotic-resistant isoleucyl-tRNA synthetase (ileS) (ARO:3000446)',

		[‘ARO:3000510’], 2, 0.001, ‘1’]			‘mupB’, [‘ARO:3000510’], 2, 0.001, ‘1’]
**oxazolidinone antibiotic (ARO:3000079)**		[‘ABC-F ATP-binding cassette ribosomal protection protein (ARO:3004469)’, ‘poxtA’, [‘ARO:3004470’], 6, 0.003, ‘0’] [‘ABC-F ATP-binding cassette ribosomal protection protein (ARO:3004469)’, ‘vgaA’, [‘ARO:3002829’], 1, 0.001, ‘0’] [‘ABC-F ATP-binding cassette ribosomal protection protein (ARO:3004469)’, ‘vgaC’, [‘ARO:3002831’], 1, 0.001, ‘0’]	linezolid (ARO:0000072)	[‘poxtA’, [‘ARO:3004470’], 6, 0.003, ‘0’]	[‘ABC-F ATP-binding cassette ribosomal protection protein (ARO:3004469)’, ‘poxtA’, [‘ARO:3004470’], 6, 0.003, ‘0’]
**penam (ARO:3000008)**		[‘methicillin resistant PBP2 (ARO:3001208)’, ‘mecA’, [‘ARO:3000617’], 35, 0.018, ‘0’]	methicillin (ARO:0000015)	[‘mecA’, [‘ARO:3000617’], 35, 0.018, ‘0’]	[‘methicillin resistant PBP2 (ARO:3001208)’, ‘mecA’, [‘ARO:3000617’], 35, 0.018, ‘0’]
**phenicol antibiotic (ARO:3000387)**		[‘ABC-F ATP-binding cassette ribosomal protection protein (ARO:3004469)’, ‘poxtA’, [‘ARO:3004470’], 6, 0.003, ‘0’] [‘ABC-F ATP-binding cassette ribosomal protection protein (ARO:3004469)’, ‘vgaA’, [‘ARO:3002829’], 1, 0.001, ‘0’] [‘ABC-F ATP-binding cassette ribosomal protection protein (ARO:3004469)’, ‘vgaC’, [‘ARO:3002831’], 1, 0.001, ‘0’]	chloramphenicol (ARO:3000385) florfenicol (ARO:3000461)	[‘poxtA’, [‘ARO:3004470’], 6, 0.003, ‘0’]	[‘ABC-F ATP-binding cassette ribosomal protection protein (ARO:3004469)’, ‘poxtA’, [‘ARO:3004470’], 6, 0.003, ‘0’]

PRJNA554461 (Yang et al. (Yang et al., 2019)).

Table F6. Comparison of UltraSEQ and WIMP Results for the Yang et al. (PRJNA554461) VAP Dataset

Platform	TPs	FNs	TNs	FPs	PPA	NPA	Accuracy

					[TP / (TP+FN)]	[TN / (TN +FP)]	
WIMP (Author)	10	2	6	7	83%	46%	64%
UltraSEQ	9	3	5	3	75%	63%	70%

Table F7. UltraSEQ's Antibiotic Genotype Profiles Agree with Phenotypic Profiles.

**Case**	AbR profile by culture*	UltraSEQ AbR summary for Identified Pathogen w/ # reads**<br>Drug class<br>Antibiotic	**Author results**
**1**	R: ticarcillin/ clavulanic acid<br>R: ceftazidime<br>I: levofloxacin<br>*NT: Tetracycline*	Cephalosporin (beta lactam): 2 reads<br><br>***Note: Others identified as well; fluoroquinolone identified in agent agnostic report***	blaTEM-4,blaTEM-112, blaTEM-157, blaACT-5, oqxB, tetC
**2**	R: methicillin<br><br>R: erythromycin, clindamycin	Penam (beta lactam): 2 reads<br>methicillin: 10 reads<br>Macrolide:<br>Roxithromycin, oleandomycin, telithromycin, spiramycin, azithromycin, clarithromycin, erythromycin, tylosin, dirithromycin: 17 reads<br>lincosamide:<br>clindamycin, lincomycin: 17 reads<br><br>***Note: several others identified as well***	mecA<br><br>ermA, erm<br><br>tet38,ant(4')-Ib, tetC, blaTEM-4
**3**	I: tetracycline	Tetracycline:<br>Tetracycline: 3 reads<br>Tigecycline: 3 reads<br><br>No Methicillin resistance identified<br>***Note: several others identified as well but all 2 reads or less; included all strains of S. aureus identified***	tetK, tet38, tetQ
**4**	R: tetracycline<br><br>R: Trimethoprim-sulfamethoxazole<br><br>R: ciprofloxacin, levofloxacin	Tetracycline:<br>Tetracycline: 14 reads<br>Minocycline, demeclocycline, oxytetracycline, chlortetracycline, doxycycline: 4 reads<br>Diaminopyrimidine:<br>Trimethoprim: 1 read	tetX<br>sul1<br>dfrA<br>acrF, pare, mdf<br>mphA, aadA5, vgaC, blaACT-5, blaACT-14, mefA, mel

		Note: Aminoglycosides and macrolides identified as well (>10 reads each); ***fluoroquinolone identified in agent agnostic report***	
**5**	S: all tested agents	None<br>No Methicillin resistance identified	None
**6**	S: all tested agents	***Note: 7 classes identified, all with 2 reads or less***<br>No Methicillin resistance identified	Tet38, blaTEM4
**7**	S: all tested agents	N/A	None
**8**	NT	***Note: 56 reads to drug efflux protein conferring resistance to multiple drug classes***	tetM, isaC, sul1, tetQ, mphA, aadA5

* R=resistant, I=intermediate, S=Susceptible, NT=Not tested; N/A = not applicable

** Only appropriate true positives and true negatives are listed (full AbR phenotype is unknown).

Results in green font indicate that for the identified pathogen, UltraSEQ identified the same antibiotic or class as the phenotype data; those in blue denote that UltraSEQ identified a closely related class; those in orange indicate that that the antibiotic was only identified in the agent agnostic report; those in *italics* were not phenotypically tested.

Illumina RNASeq Dataset: Respiratory viruses

PRJEB13360 (Graf, Flygare (Flygare et al., 2016; Graf et al., 2016)). Detailed results are provided in **Supplement D – Supplemental_File_Scores.xlsx.**

Illumina RNASeq Dataset: nasopharyngeal swabs for SARS-CoV-2 diagnosis

PRJNA634356 (Babiker et al (Babiker et al., 2020)).

Table F8. Comparison of UltraSEQ Results to Babiker et al. (KrakenUniq) and Explify

Platform	TPs	FNs	TNs	FPs	PPA [TP / (TP+FN)]	NPA [TN / (TN +FP)]	Accuracy
Author (KrakenUniq)	26	1	17	1	96%	94%	96%
Explify	20	3	16	0	86%	100%	93%
UltraSEQ	27	0	16	2	100%	89%	96%

Mixed:

de Vries et al. Dataset. (https://veb.lumc.nl/CliniMG)

Table F9. UltraSEQ Results for the de Vries Dataset Compared to Other Pipelines as Reported in (de Vries et al., 2021)

Pipeline	Positive predictive value (PPV) [%]*	PPA (Sensitivity) [%]*
**UltraSEQ**	**100**	**92**
Centrifuge	100	92
DAMIAN	100	77
DIAMOND	93	85
DNAstar	71	100
FEVIR	88	100
Genome Detective	100	85
Jovian	100	77
MetaMIC	100	77
metaMix	100	100
One Codex	100	77
RIEMS	81	85
Taxonomer	100	85
VirMet	93	92

* PPV and PPA for UltraSEQ results were determined as described in the Methods Section. PPA and PPV for all other datasets determined as reported in Supplemental Table 2 and 4, respectively in (de Vries et al., 2021).

Mixed Illumina RNASeq Dataset: In-house COVID-19 Saliva Study

Battelle (PRJNA856680).

Detailed results provided in Supplement D – Supplemental_File_Scores.xlsx.

References for Supplemental Section F

1. Babiker, A., Bradley, H. L., Stittleburg, V. D., Ingersoll, J. M., Key, A., Kraft, C. S., Waggoner, J. J., & Piantadosi, A. (2020). Metagenomic Sequencing To Detect Respiratory Viruses in Persons under Investigation for COVID-19. *J Clin Microbiol*, *59*(1). https://doi.org/10.1128/JCM.02142-20
2. de Vries, J. J. C., Brown, J. R., Fischer, N., Sidorov, I. A., Morfopoulou, S., Huang, J., Munnink, B. B. O., Sayiner, A., Bulgurcu, A., Rodriguez, C., Gricourt, G., Keyaerts, E., Beller, L., Bachofen, C., Kubacki, J., Samuel, C., Florian, L., Dennis, S., Beer, M., . . . Claas, E. C. J. (2021). Benchmark of thirteen bioinformatic pipelines for metagenomic virus diagnostics using datasets from clinical samples. *J Clin Virol*, *141*, 104908. https://doi.org/10.1016/j.jcv.2021.104908
3. Fischer, N., Indenbirken, D., Meyer, T., Lutgehetmann, M., Lellek, H., Spohn, M., Aepfelbacher, M., Alawi, M., & Grundhoff, A. (2015). Evaluation of Unbiased Next-Generation Sequencing of RNA (RNA-seq) as a Diagnostic Method in Influenza Virus-Positive Respiratory Samples. *J Clin Microbiol*, *53*(7), 2238-2250. https://doi.org/10.1128/JCM.02495-14
4. Flygare, S., Simmon, K., Miller, C., Qiao, Y., Kennedy, B., Di Sera, T., Graf, E. H., Tardif, K. D., Kapusta, A., Rynearson, S., Stockmann, C., Queen, K., Tong, S., Voelkerding, K. V., Blaschke, A., Byington, C. L., Jain, S., Pavia, A., Ampofo, K., . . . Schlaberg, R. (2016). Taxonomer: an interactive metagenomics analysis portal for universal pathogen detection and host mRNA expression profiling. *Genome Biol*, *17*(1), 111. https://doi.org/10.1186/s13059-016-0969-1
5. Graf, E. H., Simmon, K. E., Tardif, K. D., Hymas, W., Flygare, S., Eilbeck, K., Yandell, M., & Schlaberg, R. (2016). Unbiased Detection of Respiratory Viruses by Use of RNA Sequencing-Based Metagenomics: a Systematic Comparison to a Commercial PCR Panel. *J Clin Microbiol*, *54*(4), 1000-1007. https://doi.org/10.1128/JCM.03060-15
6. Hasan, M. R., Sundararaju, S., Tang, P., Tsui, K. M., Lopez, A. P., Janahi, M., Tan, R., & Tilley, P. (2020). A metagenomics-based diagnostic approach for central nervous system infections in hospital acute care setting. *Sci Rep*, *10*(1), 11194. https://doi.org/10.1038/s41598-020-68159-z
7. Miller, S., Naccache, S. N., Samayoa, E., Messacar, K., Arevalo, S., Federman, S., Stryke, D., Pham, E., Fung, B., Bolosky, W. J., Ingebrigtsen, D., Lorizio, W., Paff, S. M., Leake, J. A., Pesano, R., DeBiasi, R., Dominguez, S., & Chiu, C. Y. (2019). Laboratory validation of a clinical metagenomic sequencing assay for pathogen detection in cerebrospinal fluid. *Genome Res*, *29*(5), 831-842. https://doi.org/10.1101/gr.238170.118
8. Saha, S., Ramesh, A., Kalantar, K., Malaker, R., Hasanuzzaman, M., Khan, L. M., Mayday, M. Y., Sajib, M. S. I., Li, L. M., Langelier, C., Rahman, H., Crawford, E. D., Tato, C. M., Islam, M., Juan, Y. F., de Bourcy, C., Dimitrov, B., Wang, J., Tang, J., . . . DeRisi, J. L. (2019). Unbiased Metagenomic Sequencing for Pediatric Meningitis in Bangladesh Reveals Neuroinvasive Chikungunya Virus Outbreak and Other Unrealized Pathogens. *mBio*, *10*(6). https://doi.org/10.1128/mBio.02877-19
9. Watts, G. S., Thornton, J. E., Jr., Youens-Clark, K., Ponsero, A. J., Slepian, M. J., Menashi, E., Hu, C., Deng, W., Armstrong, D. G., Reed, S., Cranmer, L. D., & Hurwitz, B. L. (2019). Identification and quantitation of clinically relevant microbes in patient samples: Comparison of three k-mer based classifiers for speed, accuracy, and sensitivity. *PLoS Comput Biol*, *15*(11), e1006863. https://doi.org/10.1371/journal.pcbi.1006863
10. Yang, L., Haidar, G., Zia, H., Nettles, R., Qin, S., Wang, X., Shah, F., Rapport, S. F., Charalampous, T., Methe, B., Fitch, A., Morris, A., McVerry, B. J., O'Grady, J., & Kitsios, G. D. (2019). Metagenomic identification of severe pneumonia pathogens in mechanically-ventilated patients: a feasibility and clinical validity study. *Respir Res*, *20*(1), 265. https://doi.org/10.1186/s12931-019-1218-4
